# Comprehensive effects of fecal microbiota transplantation on cynomolgus macaques across various fecal conditions

**DOI:** 10.3389/fmicb.2024.1458923

**Published:** 2024-11-15

**Authors:** Philyong Kang, Gyu-Seo Bae, Eunsu Jeon, Jeonghwa Choi, Eun-Ha Hwang, Green Kim, Seung Ho Baek, Kyuyoung Shim, You Jung An, Kyung Seob Lim, Yujin Kim, Taehwan Oh, Jung Joo Hong, Wan-Kyu Lee, Seok-Hwan Kim, Bon-Sang Koo

**Affiliations:** ^1^Futuristic Animal Resource & Research Center, Korea Research Institute of Bioscience and Biotechnology, Cheongju, Republic of Korea; ^2^National Primate Research Center, Korea Research Institute of Bioscience and Biotechnology, Cheongju, Republic of Korea; ^3^KRIBB School of Bioscience, Korea University of Science & Technology (UST), Daejeon, Republic of Korea; ^4^Bacteriology Laboratory, College of Veterinary Medicine, Chungbuk National University, Cheongju, Republic of Korea; ^5^Department of Surgery, College of Medicine, Chungnam National University, Daejeon, Republic of Korea

**Keywords:** probiotics, fecal microbiota transplantation, cynomolgus monkeys, immune response, metabolic response, insulin, short chain fatty acid

## Abstract

Fecal microbiota transplantation (FMT) and probiotics therapies represent key clinical options, yet their complex effects on the host are not fully understood. We evaluated the comprehensive effects of FMT using diarrheal or normal feces, as well as probiotic therapies, on multiple anatomical sites in healthy cynomolgus macaques through colonoscopy and surgery. Our research revealed that FMT led to a partial microbiome transplantation without exhibiting the donor’s fecal clinical characteristics. Notably, FMT increased insulin and C-peptide levels in each animal according time series, regardless of fecal conditions. Immunologically, a reduction in neutrophil-to-lymphocyte ratio were exclusively observed in femoral veins of FMT group. In blood chemistry analyses, reductions in aspartate aminotransferase, blood urea nitrogen, and creatinine were observed in the femoral veins, while elevated levels of alanine aminotransferase and calcium were exclusively detected in the portal veins. These changes were not observed in the probiotic group. Also, short chain fatty acids were significantly higher increase in portal veins rather than femoral veins. Transcriptome analysis of liver tissues showed that metabolic pathways were primarily affected by both FMT and probiotics therapies. In summary, FMT therapy significantly influenced metabolic, immunologic and transcriptomic responses in normal macaque models, regardless of fecal conditions. Also, these macaque models, which utilize surgery and colonoscopy, serve as a human-like preclinical platform for evaluating long-term effects and anatomically specific responses to gut-targeted interventions, without the need for animal sacrifice.

## 1 Introduction

The gut microbiome has been extensively studied across various animal species, including humans. The gut is home to trillions of microbes, consisting of diverse bacteria, viruses, fungi, and yeast. Over 1,000 bacterial species have been identified in the gastrointestinal (GI) tract using next-generation sequencing technology (Qin et al., 2010).The characteristics of these microbiomes vary significantly from one individual to another (Lloyd-Price et al., 2016). Nevertheless, a healthy human microbiome contains a core set of microbes that play a pivotal role in maintaining the stability and resilience of the gut microbial community (Turnbaugh et al., 2007). At the phylum level, *Firmicutes*, *Bacteroidetes*, *Proteobacteria*, and *Actinobacteria* together make up over 99% of the microbiome found in the GI tract (Eckburg et al., 2005). The development of this microbiome is shaped in early life by exposure to external microbes, which is crucial for establishing a stable microbial community (Enav et al., 2022; Yatsunenko et al., 2012). Once a stable and resilient microbiome is established in later life stages, harmful non-resident microbes struggle to colonize the gastrointestinal tract because of selective pressures imposed by existing gut microbes and the host’s immune system (Lee and Chang, 2021; Lozupone et al., 2012).

The symbiotic relationship between commensal microbes and their host has been extensively elucidated through microbial-derived molecules such as short-chain fatty acids (SCFAs), tryptophan, and secondary bile acids (Canfora et al., 2015; Gheorghe et al., 2019; Wahlstrøm et al., 2016). SCFAs, including acetate, butyrate, and propionate, are produced from the fermentation of indigestible plant fibers by specific bacterial species like *Faecalibacterium prausnitzii* and *Roseburia* (Machiels et al., 2014). SCFAs serve as an energy source for the colonic epithelium, enhance gut barrier integrity by reducing gut permeability, maintain the mucosal immune system, particularly regulatory T cells (Treg cells), and increase the activity of the sympathetic nervous system while regulating lipid and glucose metabolism (He et al., 2020). The production of SCFAs can be influenced by dietary intake and the microbial composition in the GI tract. Mucolytic bacteria, especially *Akkermansia muciniphilia*, can protect the GI mucin layer, preventing bacterial invasion (Zhang et al., 2019).

Dysbiosis refers to a disturbance in gut microbes, characterized by reduced diversity, an increase in harmful bacteria, and a decrease in beneficial bacteria within the GI tract. Factors that lead to dysbiosis include antibiotic overuse, inappropriate diets (notably high-fat diets), exposure to foodborne toxins, elevated systemic inflammation, and organic pollutants (Napolitano and Covasa, 2020). Microbial imbalance has been linked to various human diseases, including neurological, hepatic, metabolic, autoimmune, cardiovascular, oncogenic, infectious, and gastrointestinal conditions (Carding et al., 2015). The intricate relationship between gut microbes and various physiological systems can be understood through the lens of multiple axes: the gut-brain, gut-liver, gut-skin, gut-immune, gut-heart, and gut-kidney axes (Anand and Mande, 2022; Sinha et al., 2021; Chen et al., 2019; Shi et al., 2017). However, direct evidence establishing whether dysbiosis is a cause or a consequence of specific diseases remains elusive. While disruption of the microbiome may not be the sole driver behind these conditions, it appears to play a contributory role in their progression via diverse mechanisms. Hence, changes in the microbiome can exert profound and complex effects on human physiology and diseases, although the precise outcomes remain largely undetermined.

Microbiome research has been actively conducted through *in vitro* and *in vivo* experiments, as well as human clinical studies. A variety of preclinical animal models, including humanized mice, honeybees, zebrafish and non-human primates have been used for these investigations (Douglas, 2019; Park and Im, 2020; Brenchley and Ortiz, 2021). The selection of animal models is often dictated by specific research objectives. However, these species possess distinct characteristics in terms of anatomy, physiology, genetics, metabolism, and immunology that set them apart from humans. For human clinical trials, time-series longitudinal studies are challenging to conduct due to the lack of data prior to disease onset (Lee and Chang, 2021). Additionally, examining tissue-specific microbiomes can be difficult in human subjects. Typically, human patients can provide only fecal samples, which primarily reflect the luminal microbiome of the descending colon (Yasuda et al., 2015). Furthermore, significant inter-individual variations exist among humans, making the interpretation of cross-sectional human microbiome studies complex (Ley et al., 2006). Currently, among preclinical animal models, old world macaque species most closely mirror the human microbiomes (Amato et al., 2019).

Within the realm of GI-targeted therapies, fecal microbiota transplantation (FMT) and probiotic therapies stand out as two prominent clinical treatment options (Suez et al., 2018). Probiotic therapy has been widely adopted for both healthy individuals and those with medical conditions. FMT presents a potential solution for otherwise intractable diseases, such as antibiotic-resistant *Clostridium difficile* infections and inflammatory bowel diseases (Drekonja et al., 2015; Colman and Rubin, 2014). Despite the evident impact of microbiome alterations on various aspects of human health, the comprehensive and direct effects of these therapies remain to be fully elucidated. In this study, we used the old world macaque, the preclinical animal model most similar to humans, to comprehensively analyze the effects of FMT and probiotic treatments from various perspectives.

## 2 Materials and methods

### 2.1 Animals

Nine female cynomolgus monkeys (*Macaca fascicularis*) aged between 6 and 10 years, were sourced from Cambodia and raised under specific pathogen-free conditions at the National Primate Research Center within the Korea Research Institute of Bioscience and Biotechnology (KRIBB). Each monkey was housed individually in indoor cages and provided with a commercial monkey feed (2050 Teklad Global 20% Protein Primate Diet, Harlan, Envigo, United Kingdom) composed of 20% crude protein, 5.4% fat, 8.1% crude fiber, and 40.1% carbohydrates. In addition to this diet, they were given seasonal fruits and had unrestricted access to water. The housing conditions adhered strictly to the minimal requirements outlined in ‘The Guide for the Care and Use of Laboratory Animals’ as proposed by the Institute for Laboratory Animal Research (ILAR) in 2010 (National Research Council, 2010). The breeding environment was automatically regulated to maintain a temperature of 24°C ± 2°C, a relative humidity of 50% ± 5%, light intensity ranging from 150 to 300 Lux, ventilation cycling 10–20 times per hour, and a 12-h light/dark cycle. Institutional non-human primate veterinary specialists confirmed that all monkeys were in good health conditions except for one monkey suffering idiopathic chronic diarrhea. Molecular tests confirmed that all animals were negative for infections with enteric pathogens, including *Campylobacter jejuni*, *Clostridium difficile*, *Salmonella* spp., *Shigella* spp., and *Yersinia enterocolitica*. Routine fecal smear examinations also showed no evidence of fecal parasite infections.

### 2.2 Animal experimental design

**Figure 1:**
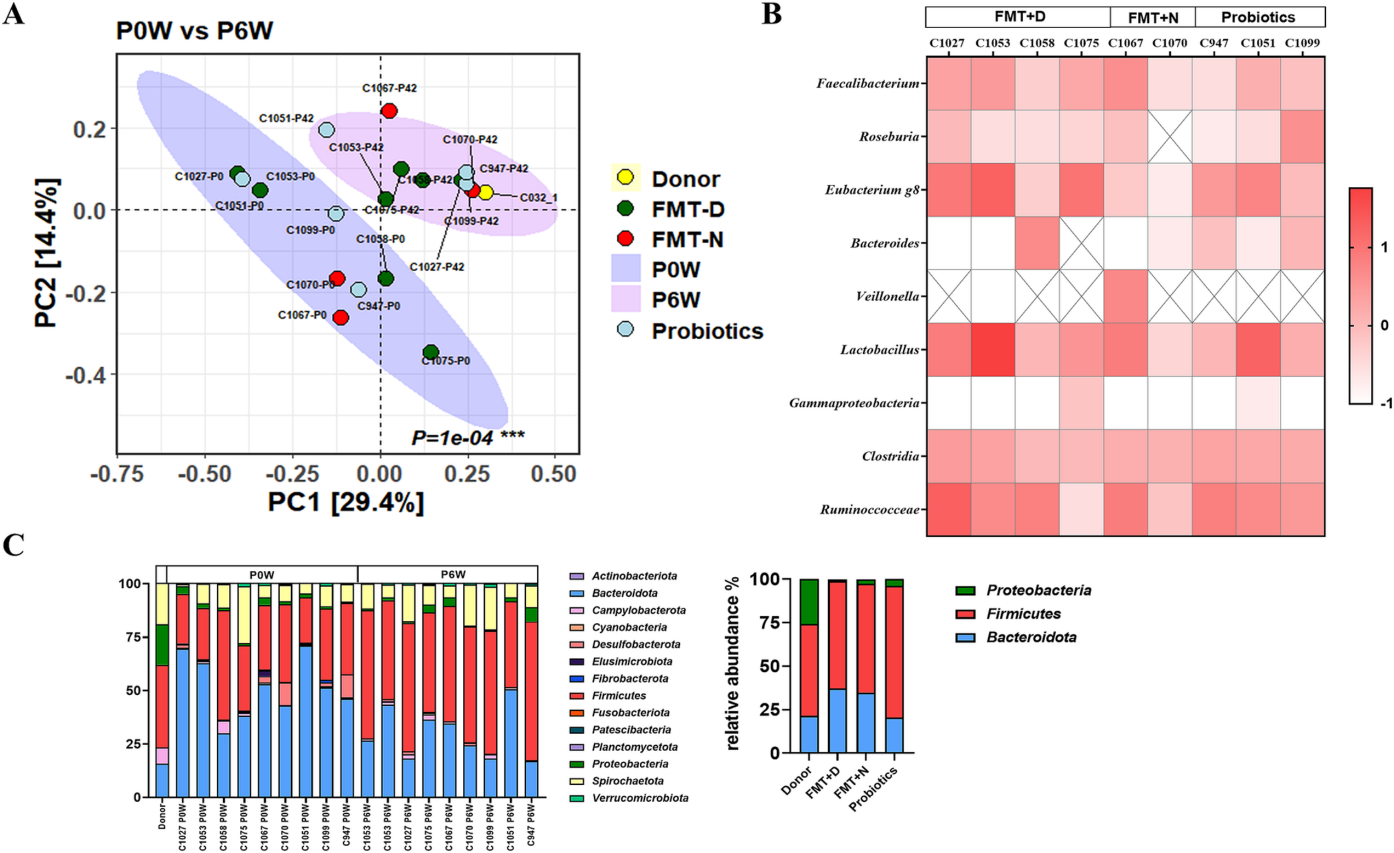
Impact of fecal Microbiota transplantation and probiotics on fecal microbial composition in recipient macaques. (A) Beta diversity analysis shows significant changes in the fecal microbiome of monkeys in both the FMT and probiotics groups between pre-and post-treatment. (B) Relative change rates of specific bacterial taxa before and after treatment. (C) Relative bacterial composition in fecal microbiome at the phylum level.

Nine healthy animals were categorized into three distinct groups. FMT + D group consisted of four animals that underwent FMT using diarrheal feces via colonoscopy. These feces were obtained from a donor monkey diagnosed with idiopathic chronic diarrhea based on clinical signs, medical imaging, and dysbiosis, without evidence of specific enteric pathogens (Koo et al., 2020). FMT + N group included two animals that received a transplant of normal feces. FMT group, which included both the FMT + D and FMT + N groups, was also assessed. Lastly, probiotics group comprised three animals who were inoculated with a six-week probiotic therapy via nasogastric tubes, using a 1ml blend of four human-origin lactic acid bacteria: *L. fermentum, L. plantarum, Leuconostoc mesenteroides*, and *Bifidobacterium breve* with over 10^9^ cfu (Table 1). Fecal sampling occurred at the outset, then at 2 and 6 weeks post-initiation of FMT and probiotic therapy. Samples, including blood from both the portal and femoral veins and liver tissues, were collected at 6 weeks post-initiation of therapy. Surgical interventions, which were executed at the beginning and 6 weeks following the FMT and probiotic treatments, facilitated the collection of liver tissue and blood from the portal veins. A Bovie electrosurgical unit was utilized during the abdominal incision and tissue collection to mitigate bleeding risks. Surgical and postoperative care were overseen by a surgical specialist alongside a non-human primate veterinary expert. Detailed specifics of the experimental design were described in Figure 1. Clinical signs were monitored daily by the breeders, and in some instances, health monitoring was performed periodically by non-human primate veterinary specialists.

**Table 1:** Detailed information of animal history and experimental designs.

Group	Animal			Experiments	
	Species	Sex	Number	Therapies	Constituents
FMT + D	*Macaca fascicularis*	Female	4	FMT	Diarrheal fresh stool
FMT + N	*Macaca fascicularis*	Female	2	FMT	Normal fresh stool
Probiotics	*Macaca fascicularis*	Female	3	Probiotics	A combination of four lactic acid bacteria

A total of nine animals were divided into three groups: FMT + D, FMT + N, and the probiotics groups. Lactic acid bacteria included *Lactobacillus fermentum*, *Lactobacillus plantarum*, *Leuconostoc mesenteroides*, and *Bifidobacterium breve*. FMT, fecal microbiota transplantation.

### 2.3 FMT therapy and surgery

We conducted FMT therapy on cynomolgus monkeys via colonoscopy using fresh feces sourced from monkeys housed in the same facility. Prior to FMT, monkeys in the designated FMT groups were treated with a combined regimen of vancomycin (15 mg/kg, IM, bid) and enrofloxacin (10 mg/kg, IM, bid) for a duration of 7 days. As a preparatory step, we administered an enema, introducing 50ml of a polyethylene glycol solution into the monkeys via nasogastric tubes on two separate occasions. We aseptically collected two distinct fecal types: normal and diarrheal. The normal fecal samples were randomly obtained from healthy monkeys and subsequently pooled in sterile plastic bags. In contrast, the diarrheal feces were obtained from a monkey exhibiting severe idiopathic chronic diarrhea, characterized by recurrent episodes of watery, bloody, and mucus-laden feces (Supplementary Figure S2). Notably, this monkey had previously been identified as having a disturbed microbiome in an earlier study (Koo et al., 2020). All fecal samples were collected within a 2-h window post-defecation. After collection, the 25g feces were combined with a sterile 40 mL PBS solution. This fecal mixture was then passed through a moistened 5-layer sterile gauze positioned in a funnel and subsequently filtered using a 70μm pore size filter. The resulting solution was administered into the transverse colon of the recipient monkeys, who were placed in a right recumbency position during the procedure. Post-transplantation, we ensured that the monkeys remained in this position for an additional 30 min to ensure optimal uptake. The entire process, spanning fecal collection to FMT therapy, was completed within a 4–6 h timeframe.

### 2.4 ^18^F-fluorodeoxyglycose (FDG) positron emission tomography (PET)/computed tomography (CT) analysis

Medical images were obtained using a PET/CT scanner (Siemens Biograph-mCT) and 18F-fluorodeoxyglucose (18F-FDG) was supplied from DuChemBio Co., Ltd. All monkeys fasted for at least 12 h before and were anesthetized with 2% isoflurane, administered via a respiratory anesthesia machine (Royal Medical) at a flow rate of 2 L/min for radiotracer injection and scan. They were given with 5.0 ± 0.5 MBq of 18F-FDG intravenously and after 1h, whole-body PET/CT scan was performed to visualize inflammation in the GI tracts for 10min/bed (5 bed) in a supine position. PET images were reconstructed to 200 × 200 matrics and 3.4 mm × 3.4 mm pixel sizes with a 3.0 mm slice thickness using a OSEM iterative algorithm (3 iterations and 21 subsets) with time of flight.

### 2.5 Microbiome analysis

Fecal samples were collected from each monkey at 0, 2, and 6 weeks following FMT and probiotic therapies. After defecation, fecal samples were collected within a 2-h window and promptly transported to the laboratory on ice. Total DNA was extracted from samples using the QIAamp Fast DNA Stool Mini kit (Qiagen). The V3 and V4 hyper-variable regions of the 16S bacterial rRNA gene were amplified from the extracted DNA using PCR. Sequence similarity thresholds for taxonomy classifications were as follows: genus (97 > X > 94.5), family (94.5 > X > 86.5), order (86.5 > X > 82), class (82 > X > 78.5), and phylum (78.5 > X > 75). The metagenomic processing data were assessed using the EZBioCloud Database, which is supported by CJ Bioscience. Bacterial composition values were examined from the phylum to genus level. Alpha diversity metrics included evenness, valid reads, operational taxonomic units (OTUs), abundance-based coverage estimator (ACE) and Chao1, Jackknife, Shannon, and Simpson indices. Beta diversity, featuring PCO analysis, was used for longitudinal comparisons between the pre-and post-treatment groups. The core microbiome was characterized as bacterial phyla accounting for at least 0.1% of the entire microbiome. We performed a comparative analysis of bacterial taxa between groups using the ANCOMBC2 package. All sequencing and analytical procedures were performed by CJ Bioscience (Korea).

### 2.6 Hematology, blood chemistry and hormone assay

Blood samples were drawn from the femoral and portal veins. Blood was collected from the femoral and portal veins at 0 and 6 weeks through a surgical procedure. Hematological values were assessed using the HEMAVET 950FS hematology analyzer (Drew Scientific Ltd.). Blood biochemical analyses were conducted using the Dri-Chem 7000i biochemistry analyzer (Fujifilm). The values for pancreatic and reproductive hormones were measured using a 96-well multiplex magnetic bead panel in the Milliplex MAP Non-Human Primate Pituitary Magnetic Bead Panel 1 (NHPPT1MG-46K) and the Milliplex MAP Non-Human Primate Metabolic Magnetic Bead Panel (NHPMHMAG-45K). Detailed information on hematological, chemistry, and hormonal values in the cynomolgus macaque are provided in Supplementary Table S1.

### 2.7 SCFAs measurement

Plasma samples from the femoral and portal veins were treated with methanol and then incubated at 30°C for 3 h in a shaking incubator. After centrifuging at 3,000 rpm for 20 min, the supernatant was passed through a 0.45-μm filter. A 1 μL aliquot of the filtered sample was injected into an Agilent GC-FID system fitted with an HP-FFAP column for chromatographic analysis. The concentrations of three SCFAs (acetic acid, butyric acid, and propionic acid) were determined using a Flame Ionization Detector. Nitrogen gas served as the carrier gas and was maintained at a consistent flow rate of 1.0 mL/min. The entire procedure was conducted by the Korea Polymer Testing and Research Institute.

### 2.8 Flow cytometry

Flow cytometric analysis of peripheral blood mononuclear cells (PBMCs) was conducted using the BD LSR Fortessa flow cytometer (BD Biosciences). PBMCs were harvested from blood samples drawn from both femoral and portal veins using Ficoll–Hypaque density gradient (Lymphoprep, Axis-Shield). Red blood cells were lysed by incubating with ACK lysis buffer (Gibco) at room temperature for 5 min. Subsequently, cell viability was assessed by staining with Fixable Viability Stain 575V (BD Biosciences) for 20 min at room temperature. For surface staining, cells were treated with the panel of antibodies for 30 min at 4°C. Post-staining, cells were rinsed with the permeabilization wash buffer and fixed using 1% paraformaldehyde. Data acquisition was executed using the LSRFortessa system (BD Bioscience) and analyzed using FlowJo v10.7.1.

### 2.9 Transcriptome analysis

Total RNA was extracted from liver tissues using TRIZOL reagent (Invitrogen) following the manufacturer’s protocol. Library construction was achieved using the TrueSeq Stranded Total RNA LT Sample Prep Kit (Gold) from Illumina Inc. following the manufacturer’s guidelines. High-throughput sequencing was conducted using paired-end sequencing (2 × 101 nt) on the NovaSeq 6000 system (Macrogen, Seoul, Korea). For functional annotation and gene-set enrichment analyses, Gene Ontology (GO) and Kyoto Encyclopedia of Genes and Genomes (KEGG) databases were used. All RNA sequencing procedures and subsequent analyses were performed by Macrogen Inc. (Seoul, Korea).

### 2.10 Statistical analysis

Statistical significance was determined using a paired t-test for paired samples. A *p*-value of less than 0.05 indicated statistical significance. All statistical analyses were conducted using GraphPad Prism software, version 8.4.3 (GraphPad Software).

### 2.11 Ethical statements

All experimental procedures were sanctioned by the Institutional Animal Care and Use Committee at Korea Research Institute of Bioscience and Biotechnology (Approval No. KRIBB-AEC-21324).

## 3 Results

### 3.1 Clinical and vital signs, and ^18^FDG-PET CT analysis

Throughout the experimental period, which extended from week 0 to week 6 after the initiation of therapies, no clinical signs were observed in any of the monkeys across all groups receiving FMT with either diarrheal (FMT + D group) or normal feces (FMT + N group), as well as probiotics (Probiotics group) (Figures 1A,B). Despite using feces with significant watery and mucous consistency from a donor monkey for the FMT in FMT + D group (Supplementary Figure S2), none of the four recipient monkeys displayed any gastrointestinal disturbances, including diarrhea, throughout the study period. No notable changes in body weight or temperature were observed in any of the monkeys during the study (Supplementary Figure S3). Consistent with the clinical observations, the ^18^FDG-PET CT analysis did not indicate any FDG uptake indicative of intestinal inflammation (Supplementary Figure S4). However, in the donor monkey suffering idiopathic chronic diarrhea (C032), extensive FDG uptake was observed throughout the entire large intestine area, including the colon and rectum (Supplementary Figure S2C) (Koo et al., 2020). All animals exhibited normal health conditions after the completion of experiments, based on clinical signs, hematology, and blood chemistry results by yearly health monitoring by Institutional veterinarians.

### 3.2 Microbiome analysis

Fecal microbiome was analyzed from fecal samples collected 0, 2, and 6 weeks post-treatment in all monkeys. In principal coordinate (PCO) analysis, statistically significant changes in the fecal microbiome were observed in both the FMT and probiotics groups between pre-and post-treatment (Figure 1A). However, full transplantation was not ultimately confirmed in any of the monkeys in FMT + D group. Also, significant differences in bacterial taxa and beta diversity among the groups were not verified (Figure 1). No significant changes were detected in alpha diversity indices, including ACE, Chao1, Jackknife, NPShannon, Shannon, or Simpson, across the FMT and probiotics groups (Supplementary Figure S5). *Bifidobacterium* spp., *Akkermansia* spp., *Butyricicoccus* spp. and *Citrobacter* spp. were rarely detected in feces of these monkeys.

### 3.3 Hematology and flow cytometry analysis

**Figure 2:**
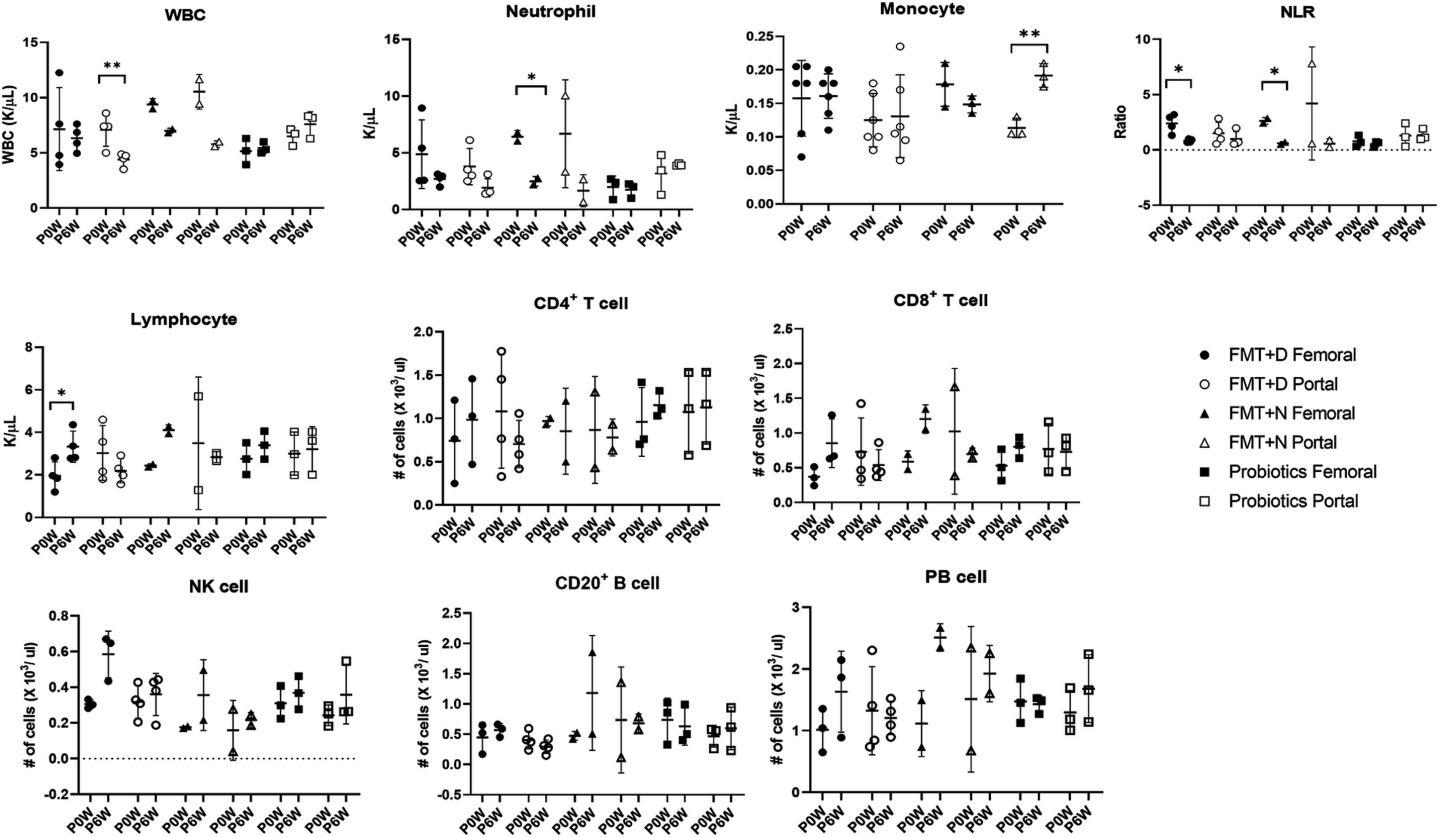
Comparative analysis of immune cell populations in femoral and portal veins of monkeys undergoing fecal microbiota transplantation with diarrheal (FMT + D) and normal (FMT + N) feces as well as probiotic groups. Monkeys undergoing fecal microbiota transplantation with diarrheal (FMT + D) and normal (FMT + N) feces, as well as those in the probiotic groups. P0W and P6W represent 0 weeks and 6 weeks post-FMT, respectively. Femoral and portal refer to the femoral and portal veins, which are anatomical sites for blood collection. The lines in the graphs indicate the mean values with standard deviation (SD). Statistically significance were determined by *t* test and indicated by *p*-values. *p* ≤ 0.05 (*); *p* ≤ 0.01 (**); *p* ≤ 0.001 (***). NLR, the neutrophil to lymphocyte ratio; WBC, white blood cells; NK cell, natural killer cell; PB cell, plasmablast.

**Figure 3:**
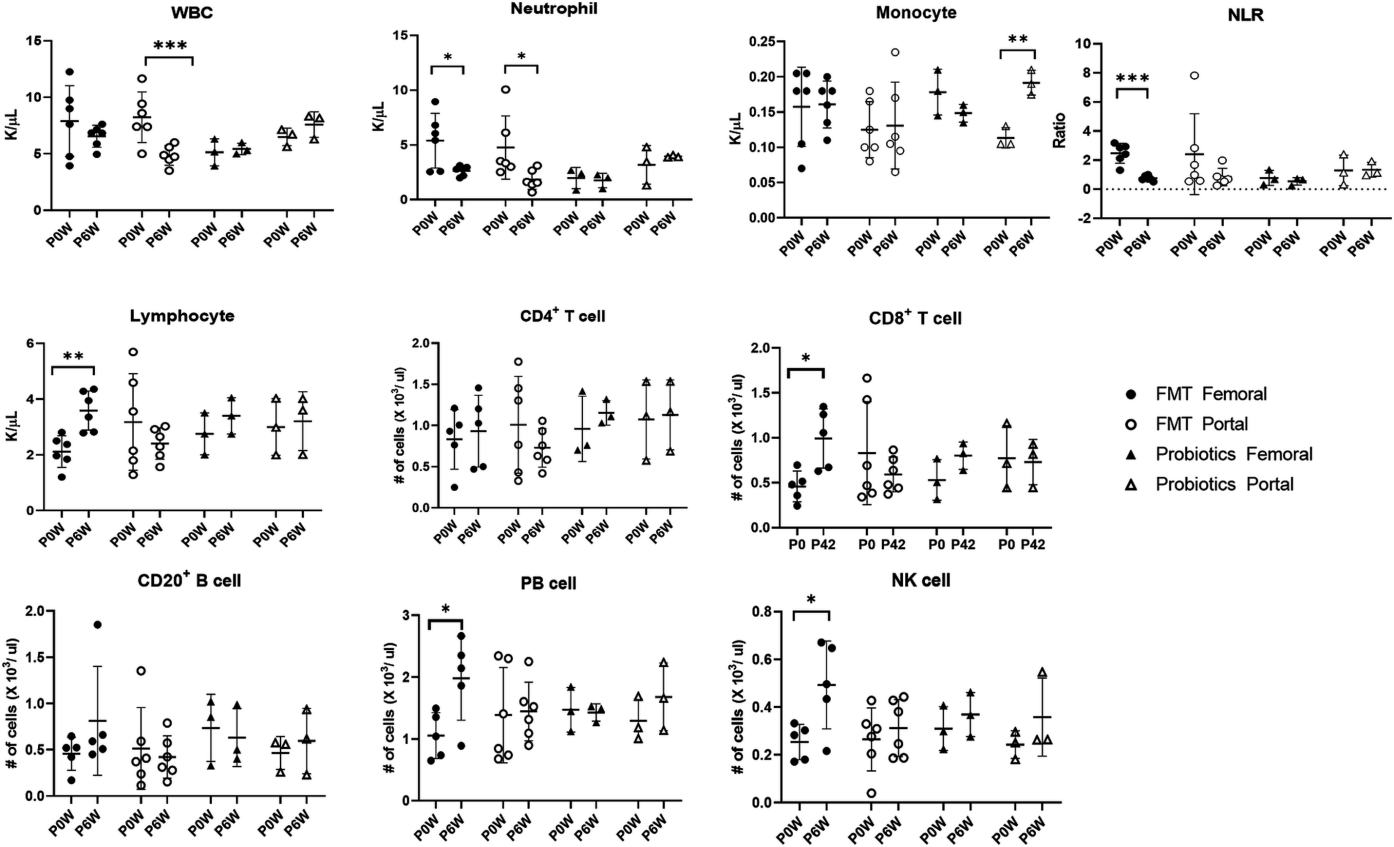
Comparative analysis of immune cell populations in femoral and portal veins of monkeys in groups of fecal microbiota transplantation (FMT) and probiotic therapies. Data in FMT group combined results from both the FMT + D and FMT + N groups. Monkeys undergoing fecal microbiota transplantation with diarrheal (FMT + D) and normal (FMT + N) feces, as well as those in the probiotic groups. P0W and P6W represent 0 weeks and 6 weeks post-FMT, respectively. Femoral and portal refer to the femoral and portal veins, which are anatomical sites for blood collection. The lines in the graphs indicate the mean values with standard deviation (SD). Statistically significance were determined by *t* test and indicated by *p*-values. *p* ≤ 0.05 (*); *p* ≤ 0.01 (**); *p* ≤ 0.001 (***). NLR, the neutrophil to lymphocyte ratio; WBC, white blood cells; NK cell, natural killer cell; PB cell, plasmablast.

Hematologic values and their subsets were assessed in all monkeys in samples from both femoral and portal veins using hematology and flow cytometry analysis. Differences were observed in hematologic values between the two anatomical sites. Hematological assessments revealed a statistically significant decrease in WBC counts in the FMT + D group, but this decrease was observed only in samples from the portal veins (Figure 2). Furthermore, reduced neutrophil counts were identified in samples from both portal and femoral veins of the FMT group which included FMT + D and FMT + N groups (Figure 3). In contrast, elevated lymphocyte counts were observed in samples from the femoral veins of the FMT group. In the Probiotic group, an increase in monocyte counts was detected only in samples from the portal veins (Figure 2, and Supplementary Figure S9). Among the lymphocyte subsets, CD8^+^, CD8^+^ T_CM_ and CD8^+^ T_EM_ cells showed a significant increase in the FMT groups (Figure 3, and Supplementary Figure S7). For B lymphocyte subsets, no notable differences were found between the FMT and Probiotic groups except for plasmablast cells (Figure 3, and Supplementary Figure S8). In the FMT groups, the counts of natural killer cells increased, but this was limited to samples from the femoral veins (Figure 3). The neutrophil to lymphocyte ratio (NLR) in peripheral blood showed a significant decrease exclusively in the femoral veins of the FMT groups (Figures 2, 3). Additionally, a rise in dendritic cell counts was identified solely in samples from the femoral veins of the FMT + D group (Supplementary Figure S9). The values of RBC, platelet and hemoglobin were not changed in all groups.

### 3.4 Hormonal response

**Figure 4:**
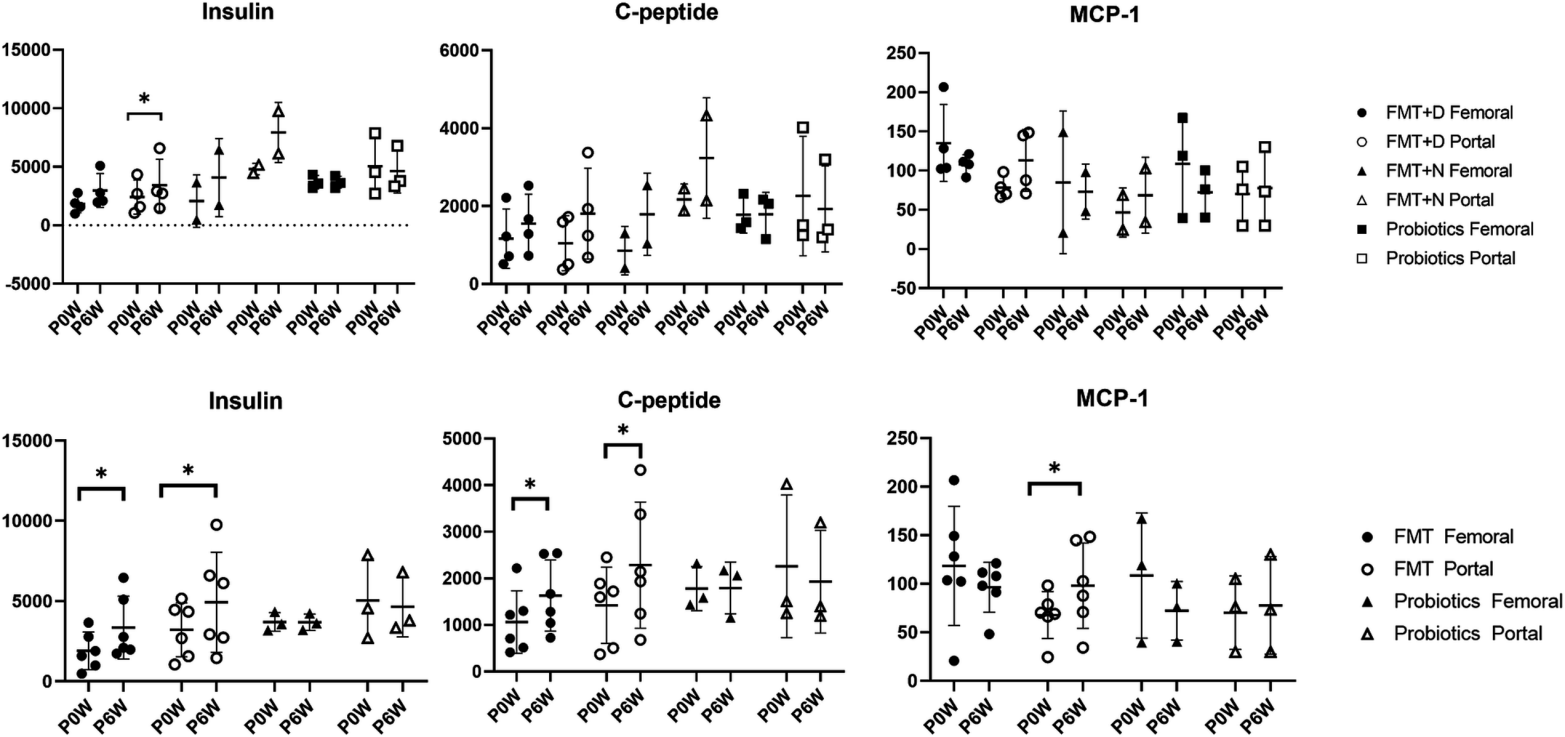
Metabolic hormone changes in femoral and portal veins of monkeys undergoing fecal microbiota transplantation and probiotic therapy. Comparative evaluations were based on differences among three groups (FMT + D, FMT + N and probiotics) and two groups (FMT and probiotics). Data in FMT group combined results from both the FMT + D and FMT + N groups. Monkeys undergoing fecal microbiota transplantation with diarrheal (FMT + D) and normal (FMT + N) feces, as well as those in the probiotic groups. P0W and P6W represent 0 weeks and 6 weeks post-FMT, respectively. Femoral and portal refer to the femoral and portal veins, which are anatomical sites for blood collection. The lines in the graphs indicate the mean values with standard deviation (SD). Statistically significance were determined by *t* test and indicated by *p*-values. *p* ≤ 0.05 (*); *p* ≤ 0.01 (**); *p* ≤ 0.001 (***); *p* ≤ 0.0001. MCP-1, monocyte chemoattractant protein-1; C-peptide, connecting peptide.

Pancreatic and reproductive hormones were measured in samples from both femoral and portal veins. In the FMT group, levels of insulin and C-peptide increased in samples from both veins, whereas no such increase was observed in the Probiotic group. Additionally, an elevated concentration of monocyte chemoattractant-1 (MCP-1) was found solely in samples from the portal veins of monkeys in the FMT group (Figure 4). There were no observed changes in the levels of gastric inhibitory polypeptide, glucagon, or pancreatic polypeptide (Supplementary Figure S10). In the FMT groups, a decrease in adrenocorticotropic hormone levels and an increase in growth hormone levels were observed in samples from the portal vein. In contrast, no reproductive hormonal changes were detected in the Probiotic group (Supplementary Figure S11).

### 3.5 Blood chemistry and SCFAs evaluation

**Figure 5:**
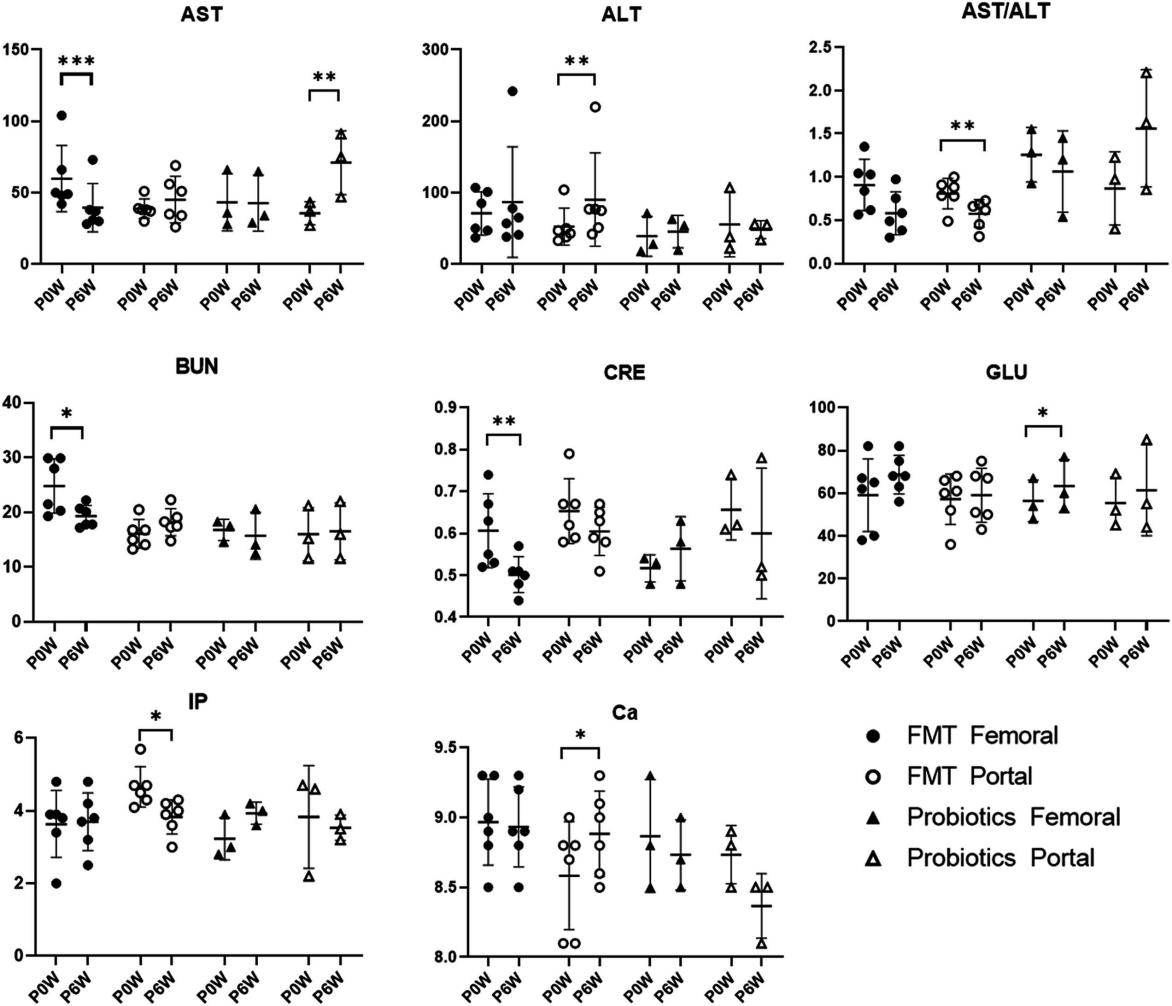
Comparative analysis of Blood chemistry results in femoral and portal veins of monkeys undergoing fecal microbiota transplantation (FMT) and probiotic therapy. Data in FMT group combined results from both the FMT + D and FMT + N groups. Monkeys undergoing fecal microbiota transplantation with diarrheal (FMT + D) and normal (FMT + N) feces, as well as those in the probiotic groups. P0W and P6W represent 0 weeks and 6 weeks post-FMT, respectively. Femoral and portal refer to the femoral and portal veins, which are anatomical sites for blood collection. The lines in the graphs indicate the mean values with standard deviation (SD). Statistically significance were determined by *t* test and indicated by *p*-values. *p* ≤ 0.05 (*); *p* ≤ 0.01 (**); *p* ≤ 0.001 (***). AST, aspartate aminotransferase; ALT, alanine aminotransferase; BUN, blood urea nitrogen; CRE, creatinine; GLU, glucose; IP, inorganic phosphate; Ca, calcium.

**Figure 6:**
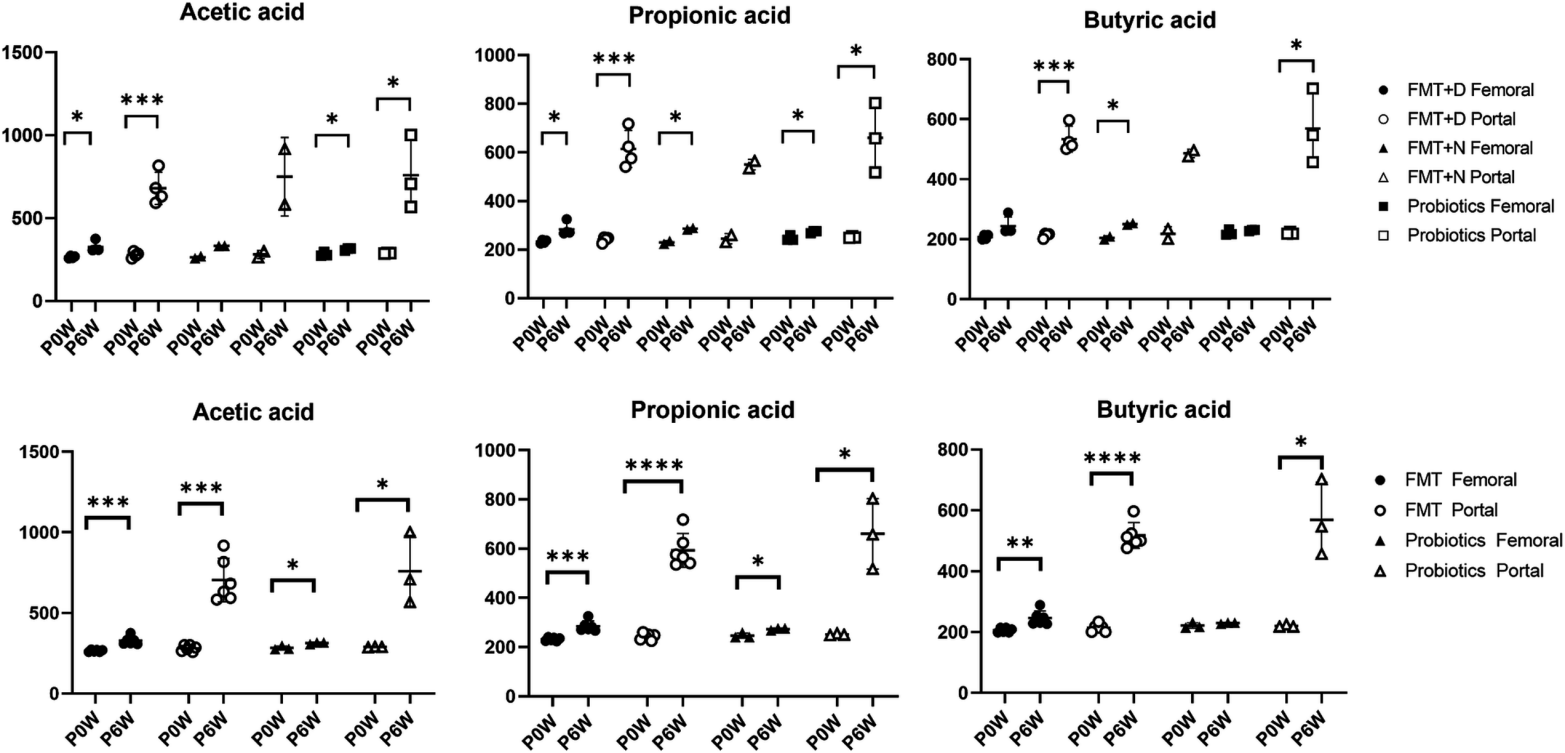
The changes of short chain fatty acids in femoral and portal veins of monkeys undergoing fecal microbiota transplantation and probiotic therapy. Short chain fatty acids include acetic acid, propionic acid, and butyric acid. Monkeys undergoing fecal microbiota transplantation with diarrheal (FMT + D) and normal (FMT + N) feces, as well as those in the probiotic groups. Comparative evaluations were based on differences among three groups (FMT + D, FMT + N and probiotics) and two groups (FMT and probiotics). Data in FMT group combined results from both the FMT + D and FMT + N groups. P0W and P6W represent 0 weeks and 6 weeks post-FMT, respectively. Femoral and portal refer to the femoral and portal veins, which are anatomical sites for blood collection. The lines in the graphs indicate the mean values with standard deviation (SD). Statistically significance were determined by *t* test and indicated by *p*-values. *p* ≤ 0.05 (*); *p* ≤ 0.01 (**); *p* ≤ 0.001 (***); *p* ≤ 0.0001.

Changes in blood chemistry and SCFAs values were observed in samples from both femoral and portal veins. In the FMT groups, aspartate aminotransferase (AST), blood urea nitrogen (BUN), and creatinine (CRE) levels decreased in samples from femoral veins. Conversely, alanine aminotransferase (ALT) and calcium (Ca) levels increased, while inorganic phosphorus (IP) levels decreased in samples from portal veins (Figure 5). For the FMT + D group, there was an increase in AST and decrease in TG values in samples from the femoral veins. Additionally, ALT values increased and albumin (ALB) values decreased in samples from the portal veins (Supplementary Figure S12). In the Probiotic group, AST values in samples from the portal veins increased, while there was a statistically significant rise in glucose (GLU) levels in samples from the femoral veins (Figure 5). All SCFAs, including acetic acid, butyric acid and propionic acid, showed increased levels in samples from both femoral and portal veins in the Probiotic group. The SCFA values were significantly higher in samples from portal veins than in those from femoral veins (Figure 6).

### 3.6 Transcriptome analysis in liver tissue

**Figure 7:**
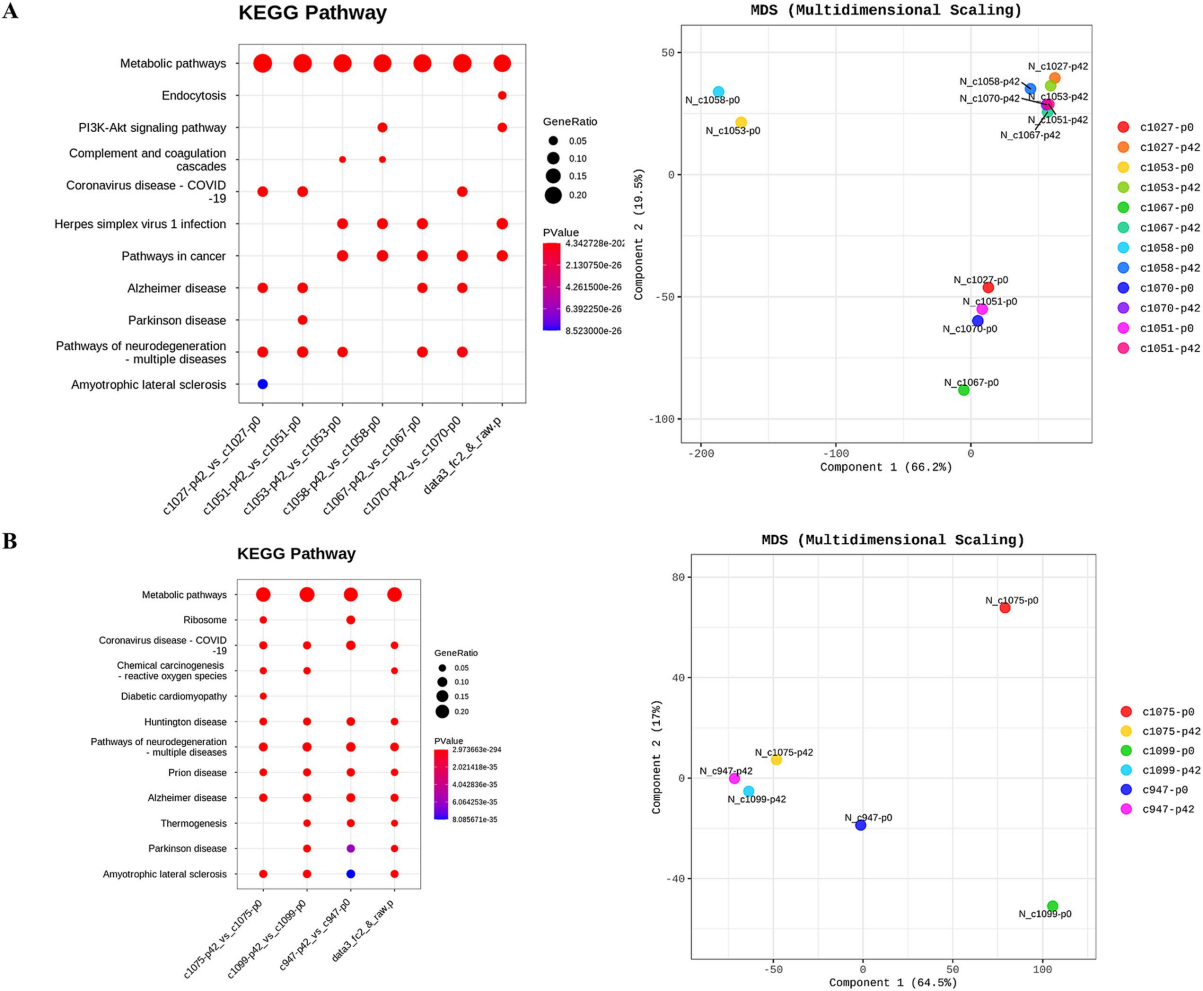
Investigation of transcriptional changes in liver tissues of monkeys undergoing fecal microbiota transplantation and probiotic therapy. Transcriptional changes were evaluated based on KEGG pathway database and multidimensional scaling analysis. (A) FMT + D group comprised C1027, C1053, C1075 and C1058 while (B) FMT + N group included C1067 and C1070, and probiotics group. P0 and P6 represent 0 weeks and 6 weeks post-FMT, respectively.

Hierarchical clustering and multi-dimensional scaling plots showed distinct clustering of RNA transcripts in liver tissues following FMT (Figure 7A) and probiotic treatments (Figure 7B). Metabolic pathways, as illustrated by KEGG pathway analyses, were predominantly influenced by both FMT and probiotics. Notably, the number of genes exhibiting up-and down-regulation varied significantly among individuals, irrespective of whether they were in the FMT or probiotic groups.

## 4 Discussion

The microbiome is recognized for its influence on both health and disease in humans. However, understanding of the intricate and complicated effects of FMT and probiotics on the healthy host remains limited. In clinical practice, feces with diverse microbiome have been used for FMT practices due to variations among individual donor stool samples. In this study, we aimed to comprehensively evaluate the effects of FMT using feces under significant different conditions, as well as probiotic therapy, across various anatomical sites in cynomolgus monkeys. This macaque model, which employs clinical techniques such as surgery and colonoscopy, could elucidate the compound effects of these gut-targeted therapies according to anatomical sites in individuals.

Changes in the microbiome have been associated with various diseases, particularly inflammatory bowel diseases (IBD) (Lee and Chang, 2021). Clinical signs were not observed in macaques until 6 weeks after the administration of FMT using diarrheal contents from a monkey with spontaneous idiopathic chronic diarrhea (Koo et al., 2020). The six-week time point was chosen because clear therapeutic effects of FMT in human patients are typically observed around 4 weeks post-treatment (Cheng et al., 2019; Fang et al., 2021). Statistically significant changes and reduced variation in the fecal microbiome were observed in both the FMT and probiotics groups between pre-and post-treatment (Figure 1A). However, full transplantation was not ultimately confirmed in any of the monkeys in FMT + D group. Also, significant differences in bacterial taxa and beta diversity among the groups were not verified (Figure 1). This finding aligns with the beta diversity results reported in human FMT studies, where fecal microbiome transplants also exhibited high variability between individuals, and none of the human patients reached the endpoint (Sokol et al., 2020). In adults, the microbiome demonstrates both stability and resilience, which may contribute to partial transplantation and inter-individual variations in the microbiome in recipient monkeys, even in the face of a high concentration of feces in the FMT (Lozupone et al., 2012; Sommer et al., 2017). To overcome the stability of gut microbiome, consequence FMT procedures were conducted for human clinical trials and significantly increased the therapeutics effects compared to single FMT procedure (Baunwall et al., 2020). Also, repeated FMT procedures were conducted for three monkeys, and no pathological signs were observed based on clinical assessments and medical diagnostics using the ^18^FDG-PET CT analysis (unpublished data). Consequently, our findings suggest that the only fecal microbiota is not a sole trigger for idiopathic chronic diarrhea in monkeys that showed similar microbiome characteristics with IBD in human (Koo et al., 2020). However, this observation does not imply that the intestinal microbiome is not a contributing factor in chronic diarrhea in both human and monkeys. It remains unclear what role the microbiome may play when combined with other factors.

Following FMT therapy, we observed a statistically significant decline in neutrophil levels in samples from both the femoral and portal veins, while elevated lymphocyte values were detected exclusively in samples from the femoral veins indicating a decrease in NLR values in healthy monkeys (Figure 3). The NLR value is a well-established prognostic biomarker for a variety of diseases, including cancer, infectious diseases, sepsis, and cardiovascular diseases (Buonacera et al., 2022). In human IBD patients, heightened neutrophil activity and extensive inflammation within the GI tracts have been identified (Herrero-Cervera et al., 2022). The observation that both decreased neutrophil levels and increased lymphocyte levels were noted in healthy individuals receiving FMT suggests that FMT may be a viable preventative strategy against various diseases in healthy populations (Goloshchapov et al., 2019). Interestingly, these preventive benefits of FMT appear to occur irrespective of the donor’s fecal conditions.

Endocrine analysis showed significant elevations in insulin and c-peptide proteins in monkeys in the FMT groups (Figure 4). These increases were evident in samples from both the femoral and portal veins, irrespective of the fecal conditions. While prior studies have highlighted the benefits of FMT therapy for both type 1 and 2 diabetes in humans, our study is the first to demonstrate in a preclinical model that FMT boosts insulin levels on healthy individuals regardless of fecal state (De Groot et al., 2021; Wu et al., 2023). The rise in MCP-1 was solely detected in samples from the portal vein. While the result was not statistically significant, we observed a trend towards declining MCP-1 values in femoral veins (Figure 4). The escalation in MCP-1 might signal an active inflammatory response and has links to various diseases (Singh et al., 2021). The observed variation in MCP-1 levels across different anatomical sites exhibited a pattern similar to blood chemistry results, where increased ALT values were noted in the portal veins, and decreased AST values were observed in the femoral veins. Consequently, FMT may be advantageous for systemic anti-inflammatory responses, yet potentially detrimental in terms of liver damage in normal people.

In both the FMT and probiotics groups, there was a dramatic increase in all SCFAs including acetate, butyrate, and propionate in samples from blood veins. In human cases, elevated levels of SCFAs have been observed following FMT procedures in patients with IBD (El-Salhy et al., 2021). Notably, the rise of SCFAs in samples from portal veins was significantly higher than those in femoral veins (Figure 6). SCFAs are generated in the colon by gut microbiota during the fermentation of non-digestible fibers and are subsequently absorbed by colonocytes and bloodstreams via portal vein to liver tissues (Bloemen et al., 2009). Due to these anatomical and physiological characteristics, the portal vein is more directly affected by changes in SCFA levels compared to the femoral vein. Therefore, the portal vein is the primary vessel directly influenced by gut microbiome–targeted treatments like FMT and probiotics (Ohtani and Hara, 2021). We predict more pronounced effects of SCFAs on liver functions through FMT and probiotics therapy via gut-liver axis, as we expected based on the results from peripheral blood. SCFAs significantly impact inflammation, immune response, anti-obesity, anti-diabetes, cardiovascular protection, hepatic protection, carcinogenesis and IBD (Visekruna and Luu, 2021; Xiong et al., 2022). Notably, butyrate promotes the differentiation of Treg cells, which play a key role in controlling inflammation (Furusawa et al., 2013). Therefore, these gut-targeted interventions may prove to be beneficial for human health from multiple aspects. These findings coincide with hepatic transcriptomic data indicating that various metabolic pathways were significantly altered following FMT and probiotic therapies (Figure 7).

The level of AST decreased in samples from the femoral veins, while ALT levels increased in samples from the portal veins (Figure 5). ALT levels are direct indicators of liver damage and are primarily found in the liver tissues (Goorden et al., 2013). On the other hand, significant concentrations of AST are not only present in the liver but also in the kidneys, skeletal muscles, and myocardium (Goorden et al., 2013). Therefore, considering anatomical and physiological characteristics of the two enzymes, ALT values from the portal vein might be the most sensitive indicator for diagnosing liver damage. We should closely monitor liver-related side effects in patients administered with FMT. With the observed simultaneous decline in CRE and BUN levels, FMT therapy could contribute to kidney health. Compared with FMT, the effects of probiotic therapy on monkeys were relatively minor. FMT has been shown to improve not only liver function in human patients with non-alcoholic fatty liver disease, alcoholic liver disease, hepatitis B virus infection, and primary sclerosing cholangitis, but also renal function in patients with chronic kidney disease (CKD) (Zhao et al., 2023; Arteaga-Muller et al., 2024). Both the therapeutic and harmful effects of FMT on liver diseases have been reported in human patients, possibly linked to increased levels of SCFAs, which can be induced by FMT and probiotics (Pant et al., 2023). Additionally, SCFAs may alleviate CKD in human patients by modulating inflammation, oxidative stress, and cellular autophagy (He et al., 2024). Notably, we observed a statistically significant increase in monocyte values in the femoral veins of monkeys treated with probiotics, an effect absent in other groups (Figure 3). *In vitro* studies have shown that *Lactobacillus(L) rhamnosus* can enhance the expression of surface molecules related to monocyte activation, thereby potentially stimulating an immune response to probiotic therapy (Rückle et al., 2023).

In this study, we investigated the systemic and multifaceted effects of FMT and probiotic therapies on various anatomical sites in healthy individuals. FMT therapy has the potential to beneficially affect immunologic and hormonal responses and SCFAs, regardless of specific fecal conditions. These host responses have also been observed in human patients undergoing the FMT procedure. Given the anatomical differences, evaluating samples from the portal veins may provide more direct insights into the interventions on the liver through the gut-liver axis. Although some invasive procedures were essential for conducting more in-depth anatomical and physiological evaluations, all animals have been maintained in normal health with appropriate veterinary care following the procedures. In conclusion, these macaque models, which utilize surgery and colonoscopy, serve as a human-like preclinical platform for evaluating longitudinal effects and anatomically specific responses to gut-targeted interventions, without the need for animal sacrifice. This report has some limitations. The effects of probiotic therapy were measured with a focus on its short-term impact. In clinical practice, probiotic therapy is typically administered over a longer period than FMT, limiting the applicability of the current findings. Furthermore, the small sample size constrains the generalizability of the results, particularly for microbiome data, due to high inter-individual variability. Future research will involve a larger number of primates to validate these findings, explore underlying mechanisms, and assess the long-term effects of the therapies. Additionally, the therapeutic potential of these interventions needs to be evaluated in primate disease models.

## Data Availability

The raw dataset in this study are available into the NCBI sequence read archive (SRA) database under accession number PRJNA1182038.
