# Supplementary material for: Comprehensive effects of fecal microbiota transplantation on cynomolgus macaques across various fecal conditions
Source: Front Microbiol. 2024 Nov 15;15:1458923. doi: 10.3389/fmicb.2024.1458923 (PMC11604628; doi:10.3389/fmicb.2024.1458923)
Supplement: Supplementary file 1 [file Supplementary_file_1.docx]

**Supplementary Materials**

**16S rRNA sequencing**

Fecal samples were collected from each monkey at 0, 2, and 6 weeks following FMT and probiotic therapy. Each monkey was housed in an individual cage. After defecation, fecal samples were collected within a 2-h window and promptly transported to the laboratory on ice. Total DNA was extracted from samples using the QIAamp Fast DNA Stool Mini kit (Qiagen, Valencia, CA, USA). The V3 and V4 hyper-variable regions of the 16S bacterial rRNA gene were amplified from the extracted DNA using PCR; this was then tagged with the Illumina NexTera barcode using the i5 sense primer and i7 antisense primer sets. PCR products were pooled together and purified using the CleanPCR system (CleanNA, Alphen aan den Rijn, The Netherlands). The quality and sizes of the amplicons were assessed using a DNA 7500 chip on a Bioanalyzer 2100 system (Agilent, Palo Alto, CA, USA). Sequencing was performed on an Illumina MiSeq Sequencing system (Illumina, San Diego, CA, USA) by CJ Bioscience Inc. (Seoul, Korea).

**Metagenomic sequence data processing and analysis**

To enhance data quality, sequences with a quality score below Q25 were excluded using the Trimmomatic 0.32 trimming tool. Pair-ended sequence data, each 250 bp in length, were assembled with PANDseq. The HMMER hmmsearch program identified non-specific amplicons, which were then excluded from subsequent analyses^31^. DUDE-Seq was used to perform denoising, while UCLUST-clustering was used to extract non-redundant reads. Bacterial taxa were classified based on the EzBioCloud database through USERCH (8.1.1861_i86linux32)^32^. Taxonomic designations were validated when sequences showed more than 97% similarity to reference data in the EzBioCloud database. Sequence similarity thresholds for taxonomy classifications were as follows: genus (97>X>94.5), family (94.5>X>86.5), order (86.5>X>82), class (82>X>78.5), and phylum (78.5>X>75). The metagenomic processing data were assessed using the EZBioCloud Database, which is supported by CJ Bioscience. Bacterial composition values were examined from the phylum to genus level. Alpha diversity metrics included evenness, valid reads, operational taxonomic units (OTUs), abundance-based coverage estimator (ACE), and Chao1, Jackknife, Shannon, and Simpson indices. Beta diversity, featuring principal coordinate (PCO) analysis, was used for longitudinal comparisons between the pre- and post-treatment groups. The core microbiome was characterized as bacterial phyla accounting for at least 0.1% of the entire microbiome. All processing and analytical procedures were performed by CJ Bioscience (Seoul, Korea).

**Blood sampling**

After an overnight fast, monkeys were anesthetized using ketamine sodium (10 mg/kg) administered via the intramuscular route. Blood samples were drawn from the femoral and portal veins. Blood was collected from the femoral veins at 0, 2, and 6 weeks of therapy and from the portal veins at 0 and 6 weeks through a surgical procedure. Immediately after blood collection, each blood sample was transferred to tubes containing dipotassium ethylenediaminetetraacetic acid (EDTA-K2) for hematological and hormonal analyses and into sodium heparin tubes for blood chemistry. Plasma was separated from blood in the EDTA-K2 tubes by centrifugation at 1,600x *g* for 15 min.

**Flow cytometry**

Flow cytometric analysis of peripheral blood mononuclear cells (PBMCs) was conducted using the BD LSR Fortessa flow cytometer (BD Biosciences). PBMCs were harvested from blood samples drawn from both femoral and portal veins using Ficoll-Hypaque density gradient (Lymphoprep, Axis-Shield). Red blood cells were lysed by incubating with ACK lysis buffer (Gibco) at room temperature for 5 min. Subsequently, cell viability was assessed by staining with Fixable Viability Stain 575V (BD Biosciences) for 20 min at room temperature. For surface staining, cells were treated with the following panel of antibodies for 30 min at 4°C: CD3 (Alexa Fluor 700; BD Bioscience), CD20 (APC/Cyanine7; BD Bioscience), CD27 (PE/Cyanine7; BD Bioscience), IgD (PE; Southern Biotech), NKG2A (APC; Beckman), CD123 (PE/Cyanine7; BD Bioscience), CD14 (PE/Cyanine7; Biolegend), CD4 (PE/Cyanine5; Biolegend), HLA-DR (Texas-Red; Invitrogen), CD80 (PE; BD Bioscience), CD11c (APC; Biolegend), CD16 (APC; BD Bioscience), CD4 (V500; BD Bioscience), CD8 (V450; BD Bioscience), CD95 (PE/Cyanine5; BD Bioscience), CD28 (PE/TexRed; Beckman Coulter, Brea, CA, USA). Post-staining, cells were rinsed with the permeabilization wash buffer and fixed using 1% paraformaldehyde. Data acquisition was executed using the LSRFortessa system (BD Bioscience) and analyzed using FlowJo v10.7.1.

**Transcriptome analysis**

Total RNA was extracted from liver tissues using TRIZOL reagent (Invitrogen) following the manufacturer’s protocol. The RNA quantity was assessed by the picogreen method using Victor 3 fluorometry. The integrity of the total RNA was confirmed using an Agilent Technologies 2100 Bioanalyzer, and samples with an RNA integrity number (RIN) value of 7 or higher were used for analyses.

Library construction was achieved using the TrueSeq Stranded Total RNA LT Sample Prep Kit (Gold) from Illumina Inc. (San Diego, USA) following the manufacturer's guidelines. High-throughput sequencing was conducted using paired-end sequencing (2 X 101 nt) on the NovaSeq 6000 system (Macrogen, Seoul, Korea). Quality assessment of the raw data was executed using FastQX v0.11.7. The sequencing reads underwent trimming with Trimmomatic 0.38 and were subsequently mapped to the reference genome with the HISAT2 software. The alignment data served as the foundation for transcript assembly using StringTie v2.1.3b, facilitating the estimation of transcript abundance and identification of differentially expressed genes (DEGs). To identify DEGs in serial liver tissues within each subject, normalization values of FPKM/RPKM and TPM were employed. For functional annotation and gene-set enrichment analyses, Gene Ontology (GO) and Kyoto Encyclopedia of Genes and Genomes (KEGG) databases were used. All RNA sequencing procedures and subsequent analyses were performed by Macrogen Inc. (Seoul, Korea).
