# Supplementary material for: Comprehensive effects of fecal microbiota transplantation on cynomolgus macaques across various fecal conditions
Source: Front Microbiol. 2024 Nov 15;15:1458923. doi: 10.3389/fmicb.2024.1458923 (PMC11604628; doi:10.3389/fmicb.2024.1458923)
Supplement: Supplementary file 2 [file Presentation_1.pptx]

## Slide 1
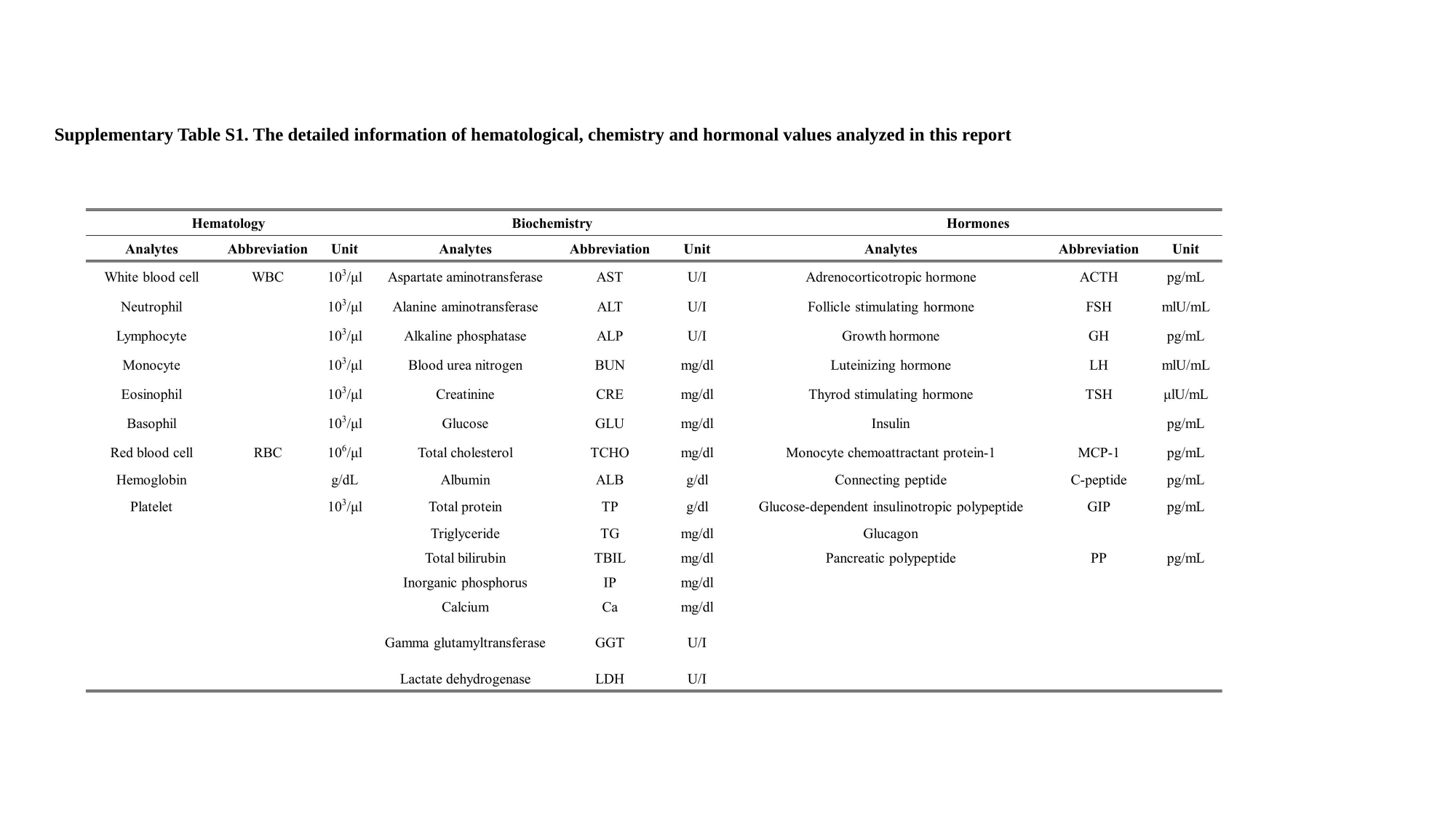

Supplementary Table S1. The detailed information of hematological, chemistry and hormonal values analyzed in this report

## Slide 2
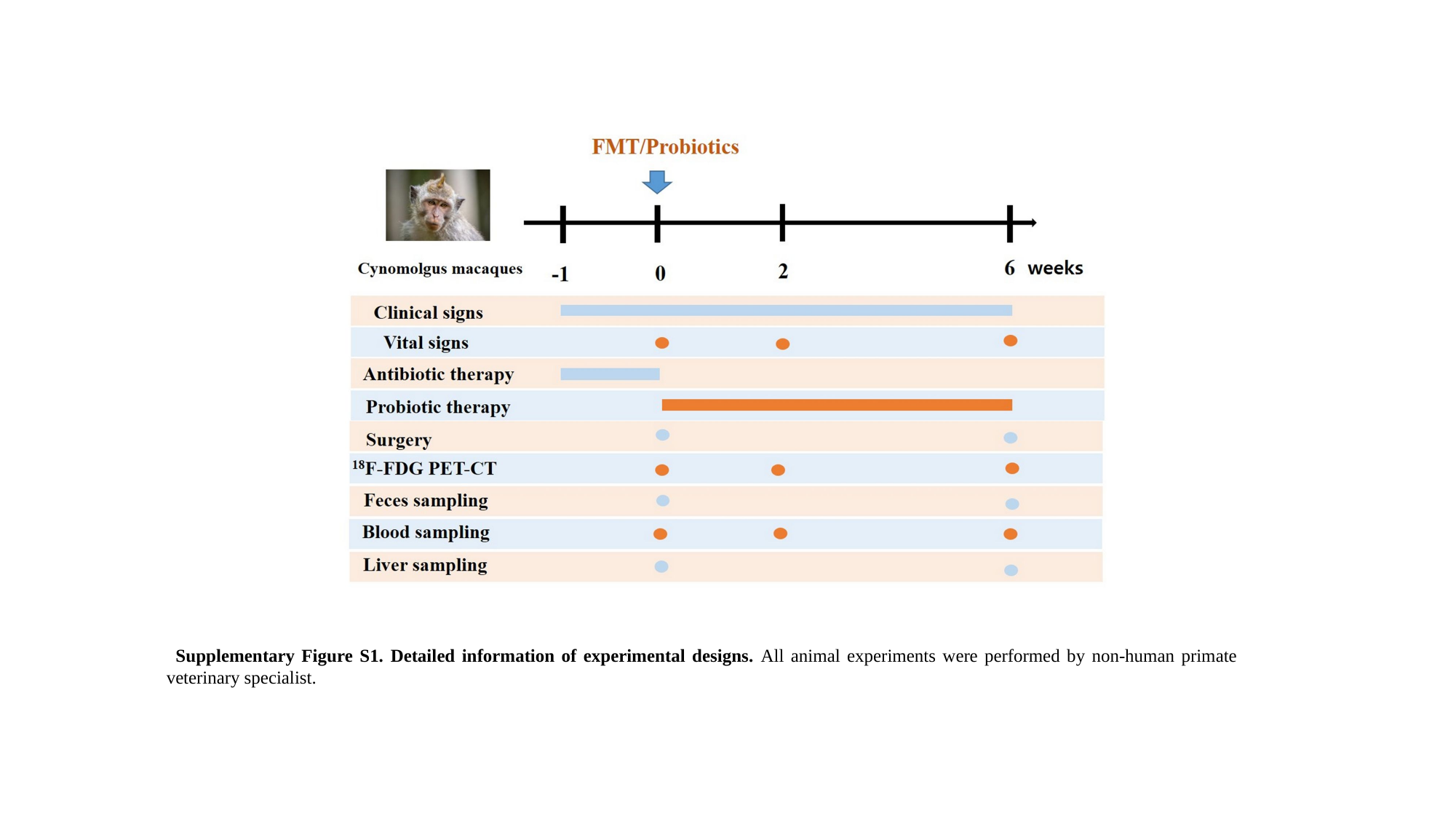

Supplementary Figure S1. Detailed information of experimental designs. All animal experiments were performed by non-human primate veterinary specialist.

## Slide 3
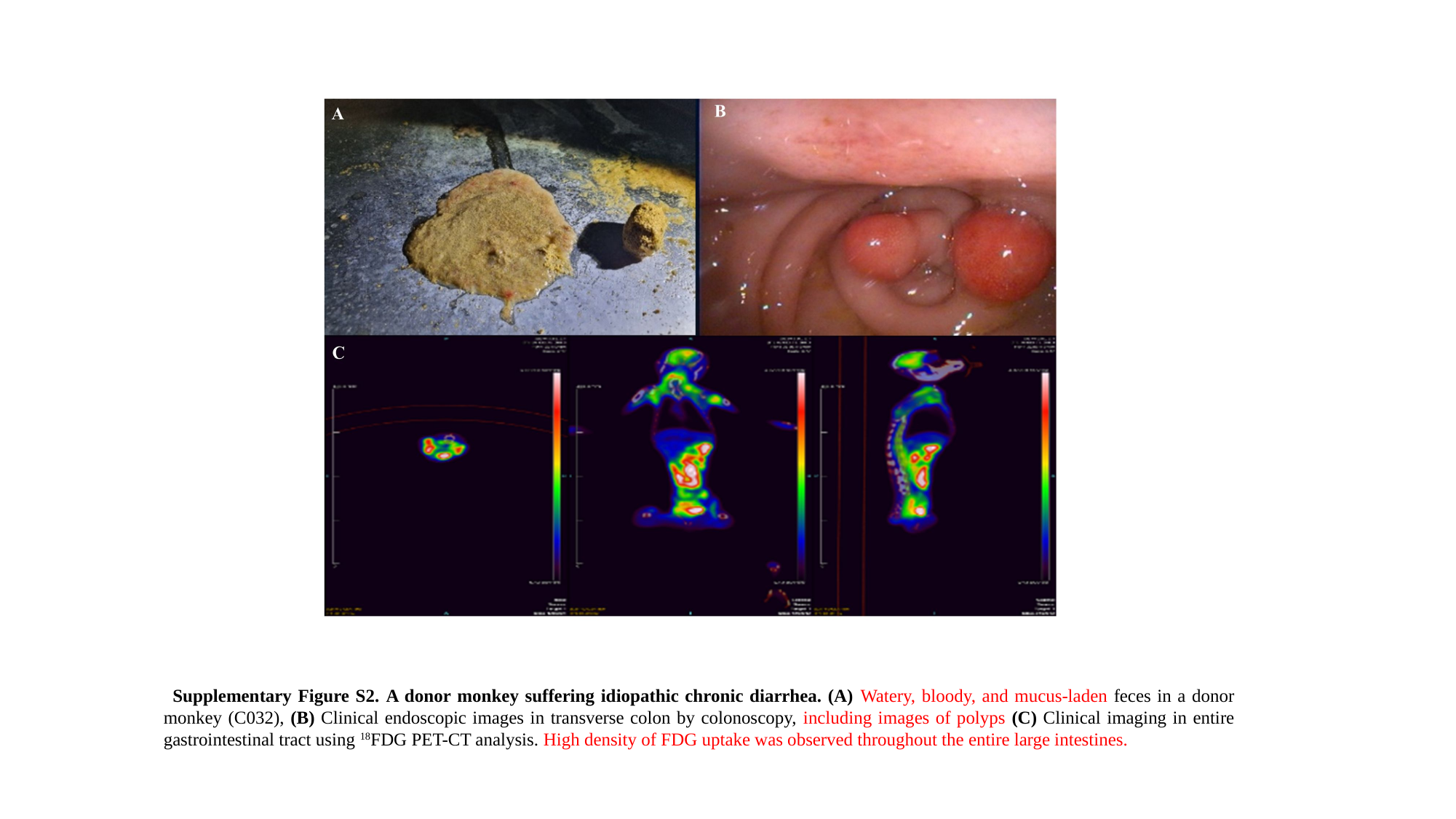

Supplementary Figure S2. A donor monkey suffering idiopathic chronic diarrhea. (A) Watery, bloody, and mucus-laden feces in a donor monkey (C032), (B) Clinical endoscopic images in transverse colon by colonoscopy, including images of polyps (C) Clinical imaging in entire gastrointestinal tract using 18FDG PET-CT analysis. High density of FDG uptake was observed throughout the entire large intestines.

## Slide 4
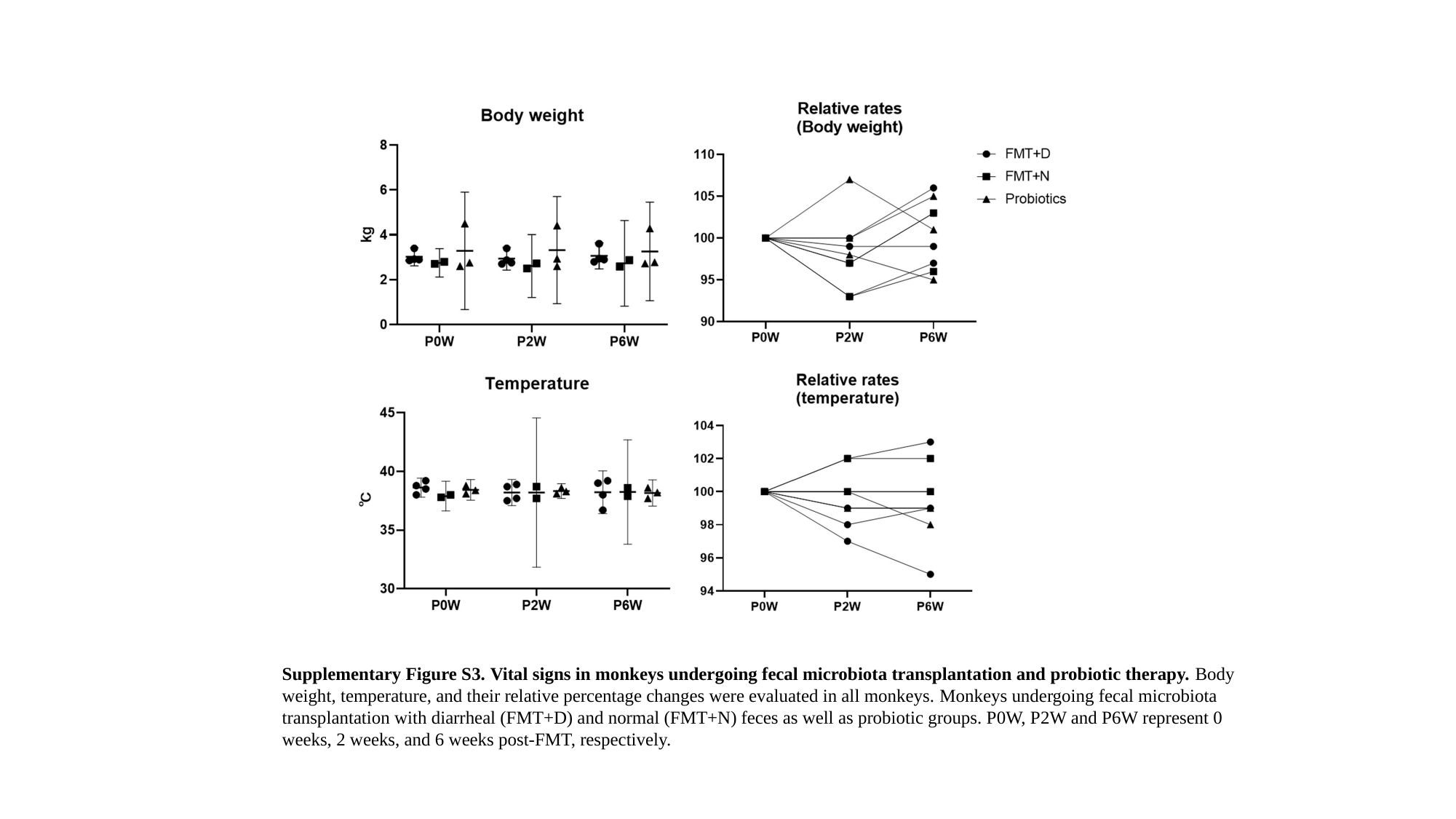

Supplementary Figure S3. Vital signs in monkeys undergoing fecal microbiota transplantation and probiotic therapy. Body weight, temperature, and their relative percentage changes were evaluated in all monkeys. Monkeys undergoing fecal microbiota transplantation with diarrheal (FMT+D) and normal (FMT+N) feces as well as probiotic groups. P0W, P2W and P6W represent 0 weeks, 2 weeks, and 6 weeks post-FMT, respectively.

## Slide 5
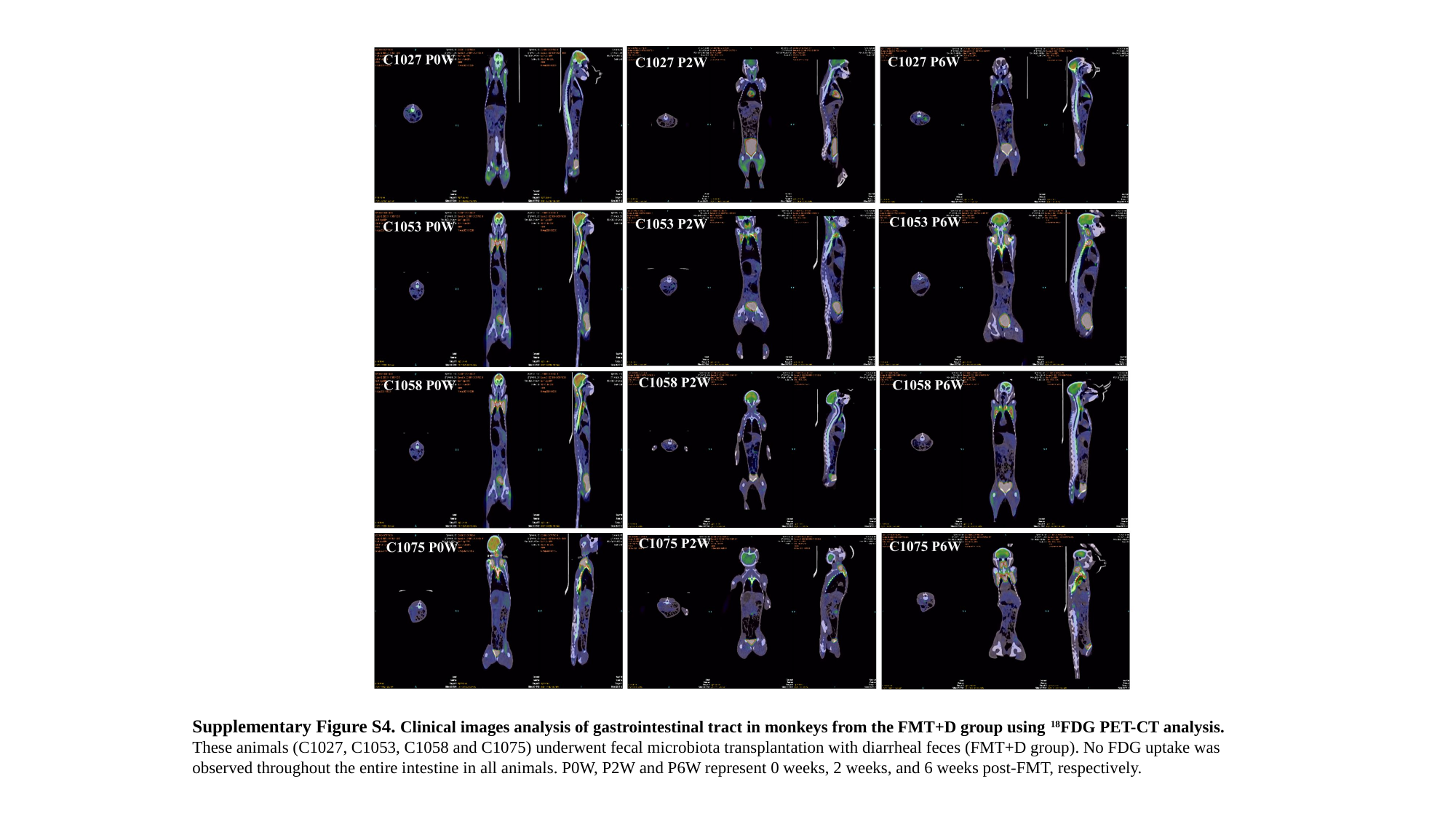

Supplementary Figure S4. Clinical images analysis of gastrointestinal tract in monkeys from the FMT+D group using 18FDG PET-CT analysis. These animals (C1027, C1053, C1058 and C1075) underwent fecal microbiota transplantation with diarrheal feces (FMT+D group). No FDG uptake was observed throughout the entire intestine in all animals. P0W, P2W and P6W represent 0 weeks, 2 weeks, and 6 weeks post-FMT, respectively.

## Slide 6
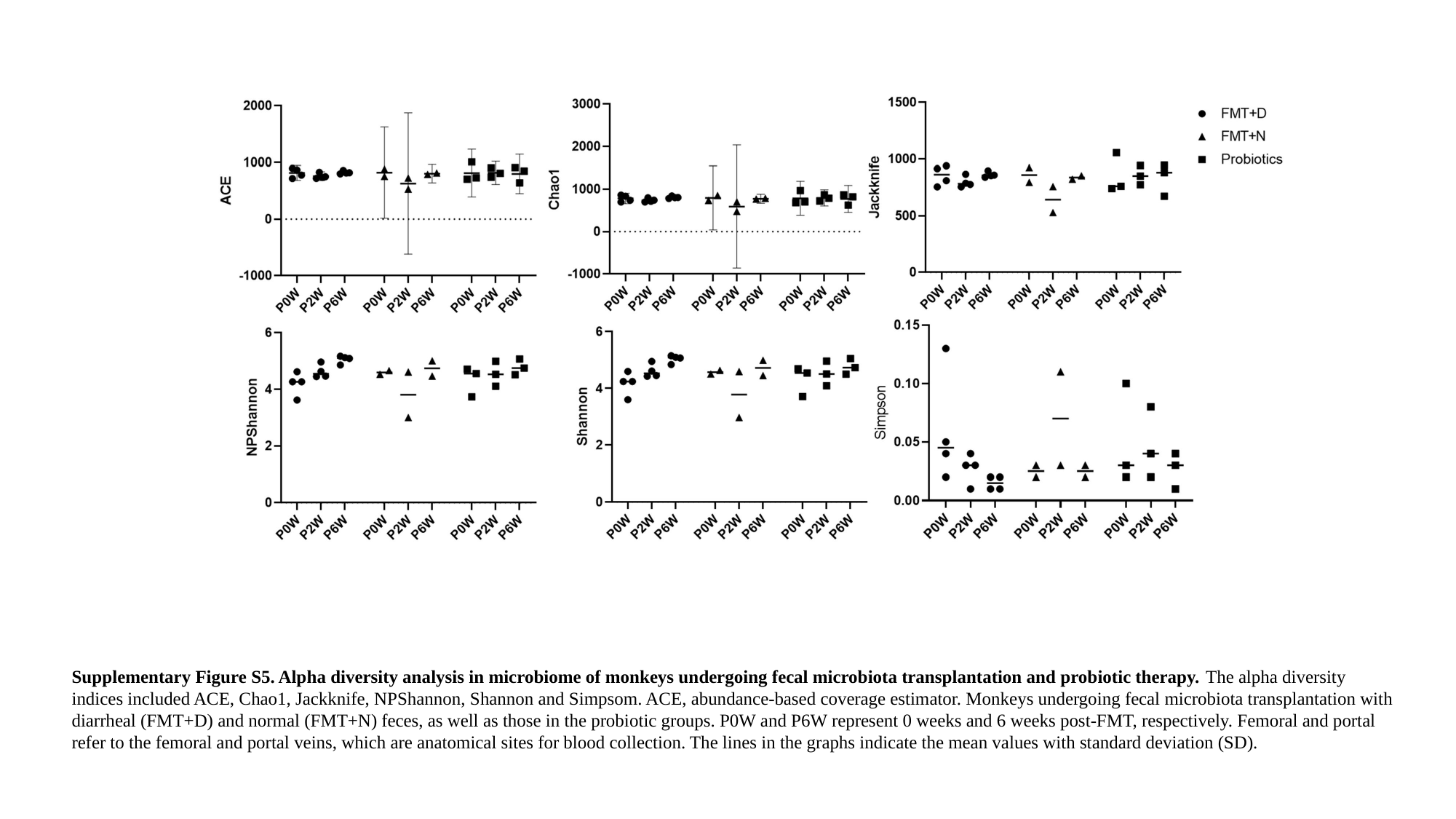

Supplementary Figure S5. Alpha diversity analysis in microbiome of monkeys undergoing fecal microbiota transplantation and probiotic therapy. The alpha diversity indices included ACE, Chao1, Jackknife, NPShannon, Shannon and Simpsom. ACE, abundance-based coverage estimator. Monkeys undergoing fecal microbiota transplantation with diarrheal (FMT+D) and normal (FMT+N) feces, as well as those in the probiotic groups. P0W and P6W represent 0 weeks and 6 weeks post-FMT, respectively. Femoral and portal refer to the femoral and portal veins, which are anatomical sites for blood collection. The lines in the graphs indicate the mean values with standard deviation (SD).

## Slide 7
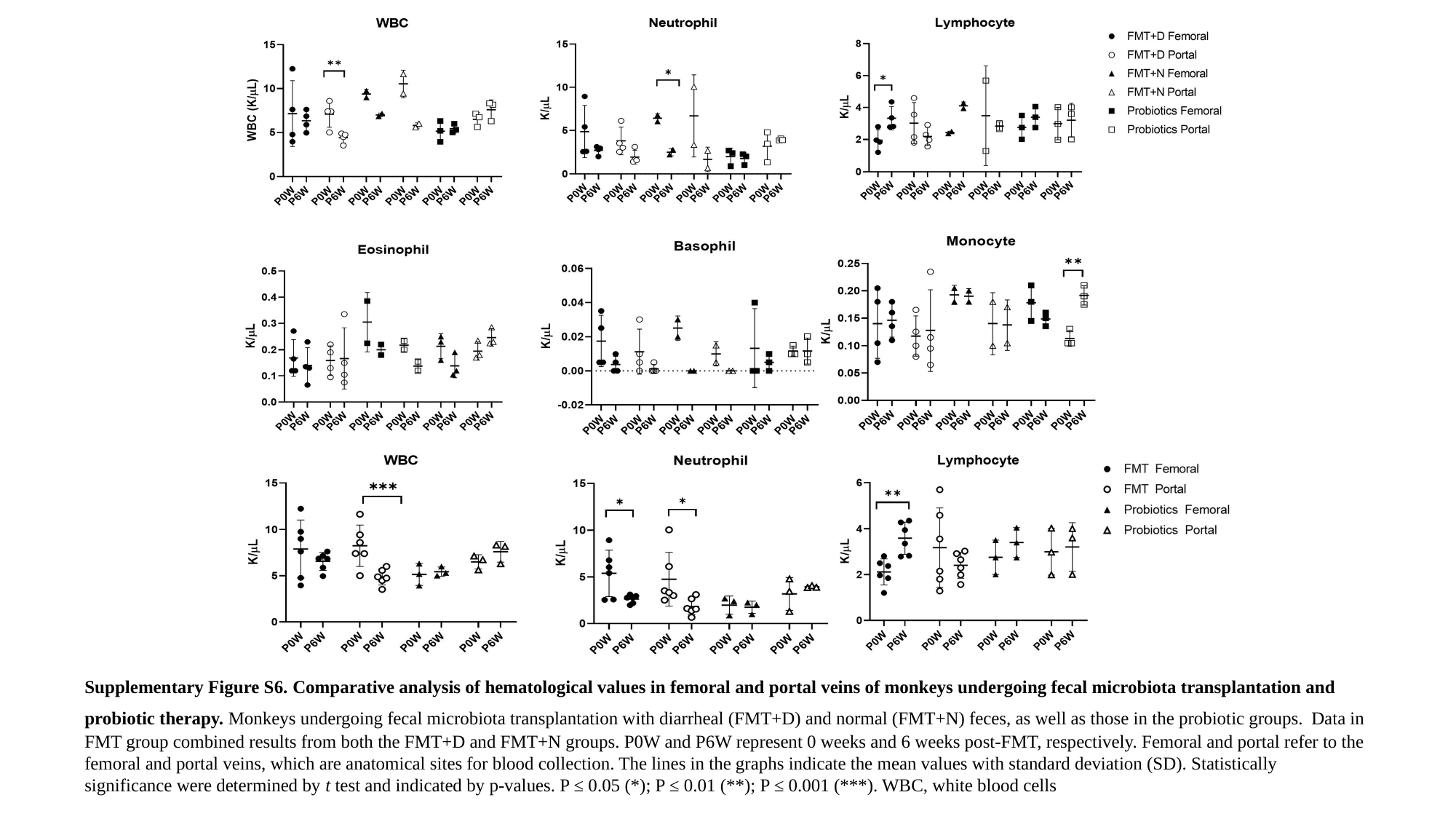

Supplementary Figure S6. Comparative analysis of hematological values in femoral and portal veins of monkeys undergoing fecal microbiota transplantation and probiotic therapy. Monkeys undergoing fecal microbiota transplantation with diarrheal (FMT+D) and normal (FMT+N) feces, as well as those in the probiotic groups. Data in FMT group combined results from both the FMT+D and FMT+N groups. P0W and P6W represent 0 weeks and 6 weeks post-FMT, respectively. Femoral and portal refer to the femoral and portal veins, which are anatomical sites for blood collection. The lines in the graphs indicate the mean values with standard deviation (SD). Statistically significance were determined by t test and indicated by p-values. P ≤ 0.05 (*); P ≤ 0.01 (**); P ≤ 0.001 (***). WBC, white blood cells

## Slide 8
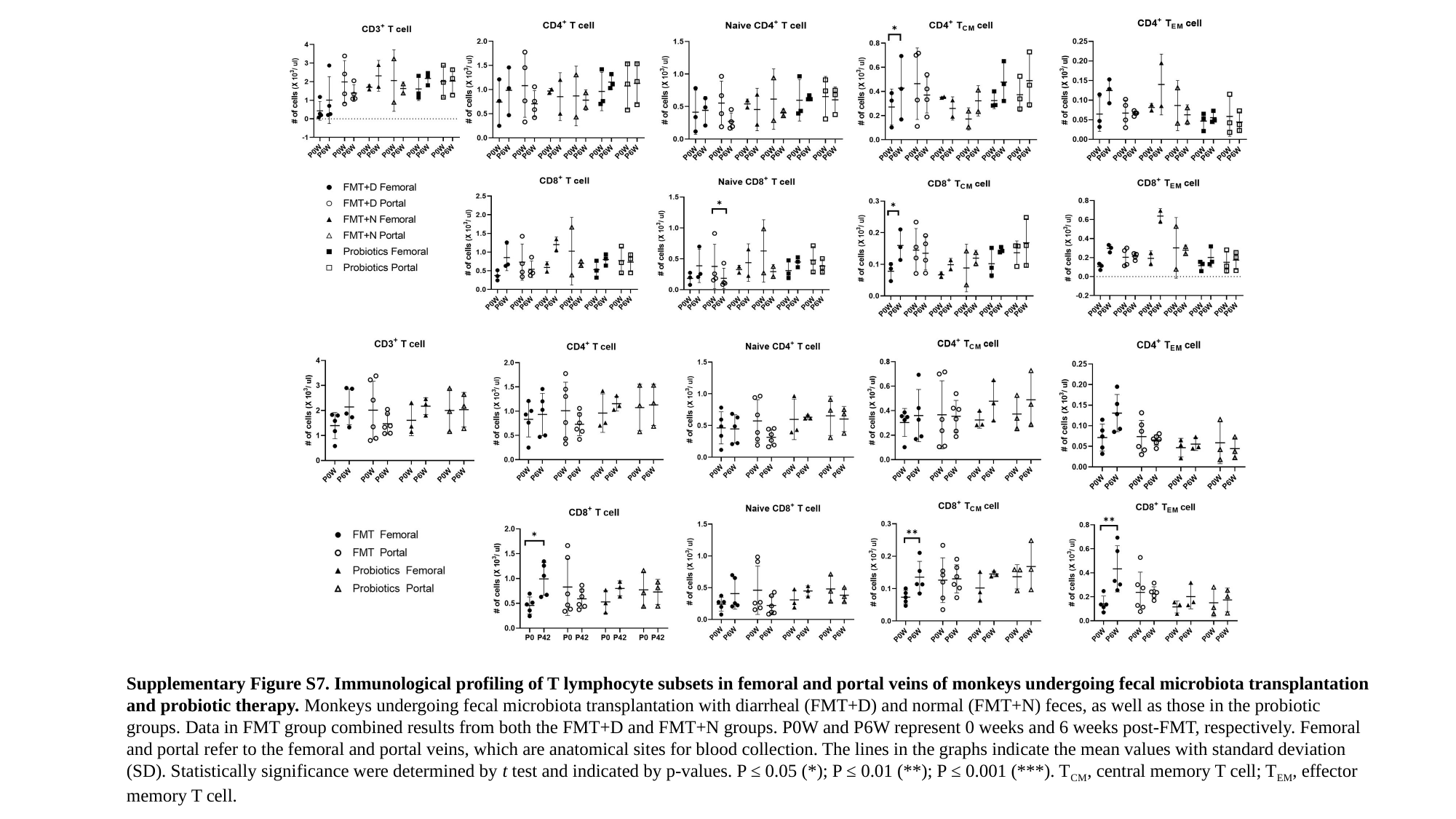

Supplementary Figure S7. Immunological profiling of T lymphocyte subsets in femoral and portal veins of monkeys undergoing fecal microbiota transplantation and probiotic therapy. Monkeys undergoing fecal microbiota transplantation with diarrheal (FMT+D) and normal (FMT+N) feces, as well as those in the probiotic groups. Data in FMT group combined results from both the FMT+D and FMT+N groups. P0W and P6W represent 0 weeks and 6 weeks post-FMT, respectively. Femoral and portal refer to the femoral and portal veins, which are anatomical sites for blood collection. The lines in the graphs indicate the mean values with standard deviation (SD). Statistically significance were determined by t test and indicated by p-values. P ≤ 0.05 (*); P ≤ 0.01 (**); P ≤ 0.001 (***). TCM, central memory T cell; TEM, effector memory T cell.

## Slide 9
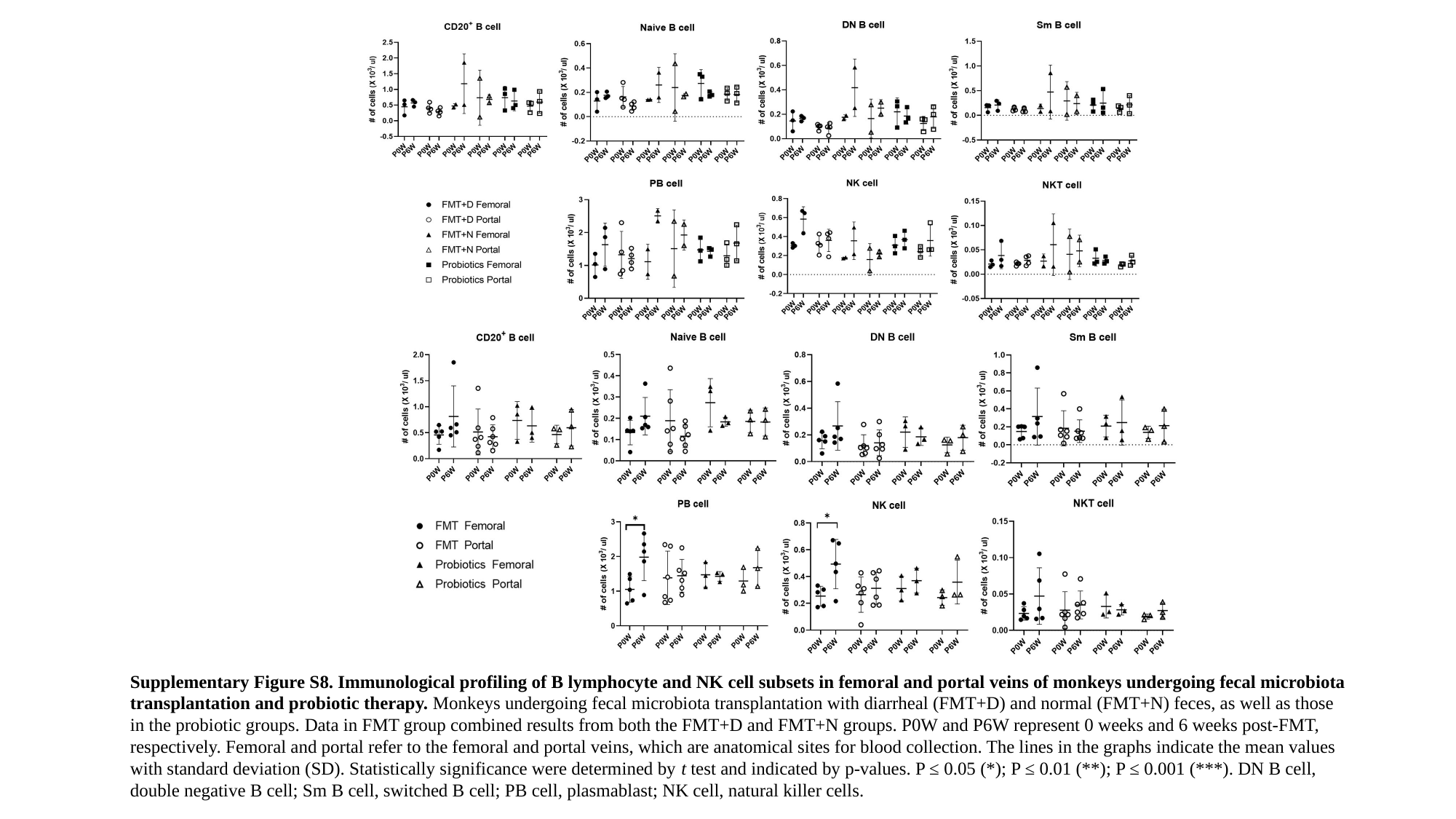

Supplementary Figure S8. Immunological profiling of B lymphocyte and NK cell subsets in femoral and portal veins of monkeys undergoing fecal microbiota transplantation and probiotic therapy. Monkeys undergoing fecal microbiota transplantation with diarrheal (FMT+D) and normal (FMT+N) feces, as well as those in the probiotic groups. Data in FMT group combined results from both the FMT+D and FMT+N groups. P0W and P6W represent 0 weeks and 6 weeks post-FMT, respectively. Femoral and portal refer to the femoral and portal veins, which are anatomical sites for blood collection. The lines in the graphs indicate the mean values with standard deviation (SD). Statistically significance were determined by t test and indicated by p-values. P ≤ 0.05 (*); P ≤ 0.01 (**); P ≤ 0.001 (***). DN B cell, double negative B cell; Sm B cell, switched B cell; PB cell, plasmablast; NK cell, natural killer cells.

## Slide 10
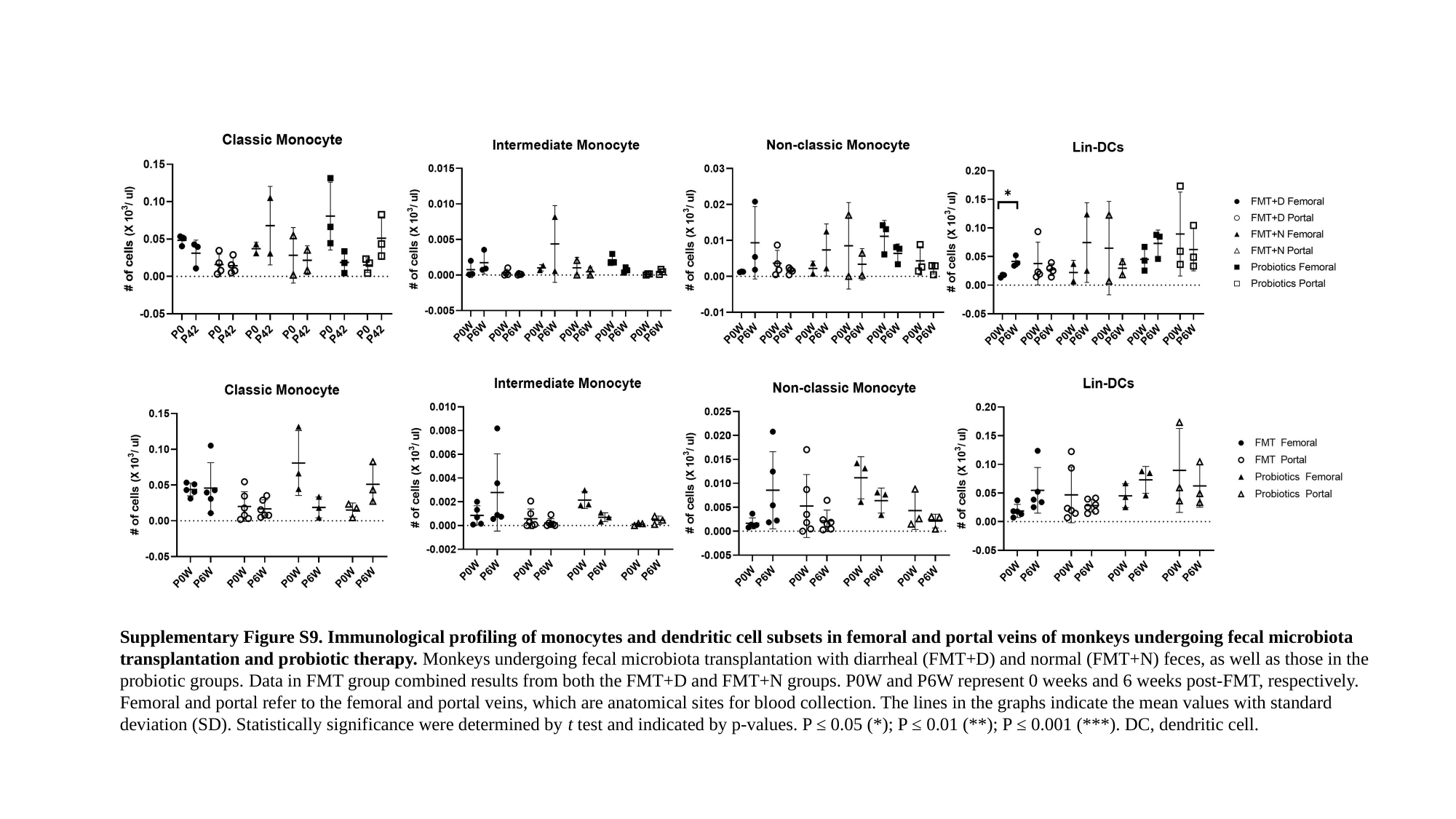

Supplementary Figure S9. Immunological profiling of monocytes and dendritic cell subsets in femoral and portal veins of monkeys undergoing fecal microbiota transplantation and probiotic therapy. Monkeys undergoing fecal microbiota transplantation with diarrheal (FMT+D) and normal (FMT+N) feces, as well as those in the probiotic groups. Data in FMT group combined results from both the FMT+D and FMT+N groups. P0W and P6W represent 0 weeks and 6 weeks post-FMT, respectively. Femoral and portal refer to the femoral and portal veins, which are anatomical sites for blood collection. The lines in the graphs indicate the mean values with standard deviation (SD). Statistically significance were determined by t test and indicated by p-values. P ≤ 0.05 (*); P ≤ 0.01 (**); P ≤ 0.001 (***). DC, dendritic cell.

## Slide 11
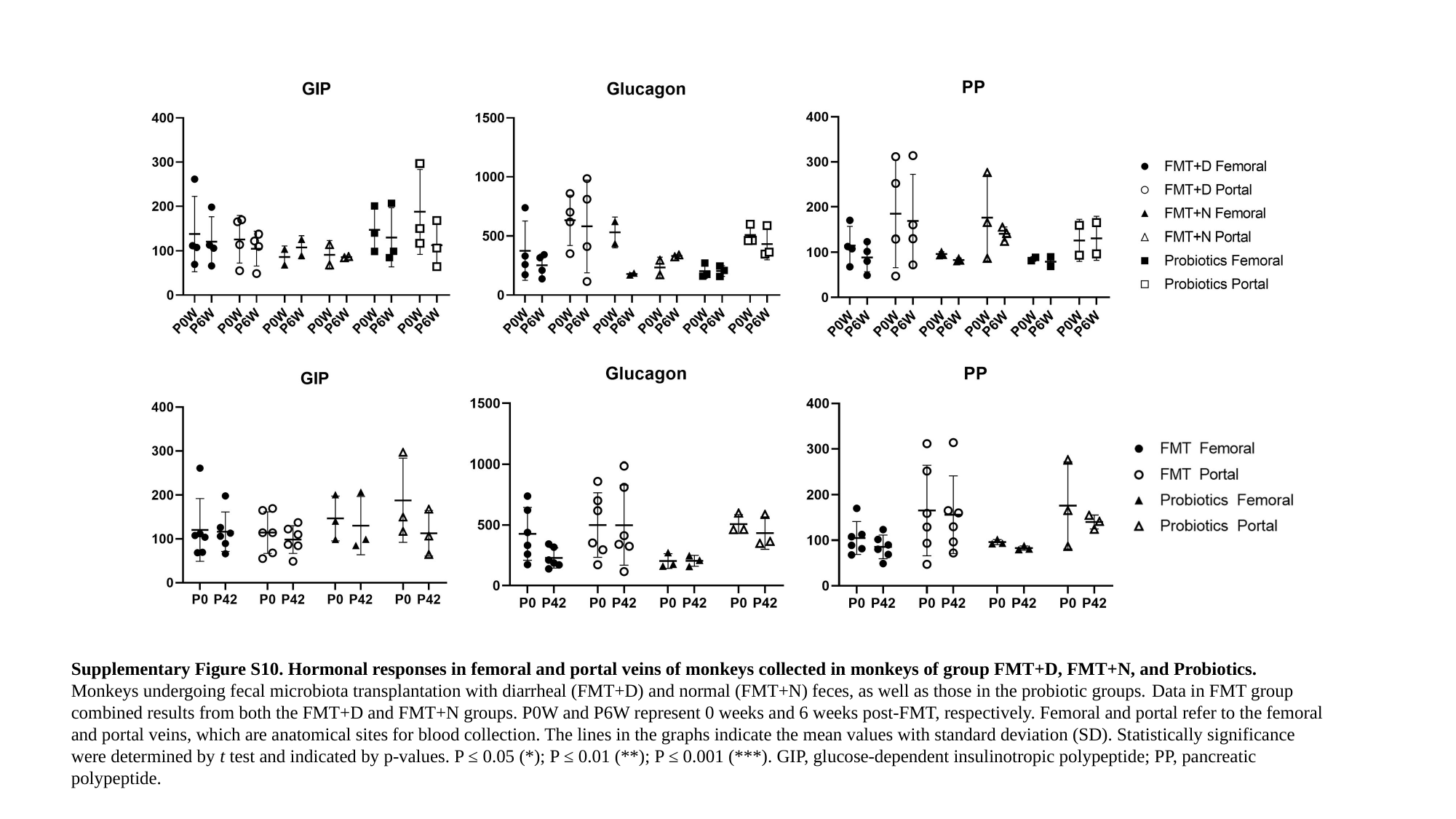

Supplementary Figure S10. Hormonal responses in femoral and portal veins of monkeys collected in monkeys of group FMT+D, FMT+N, and Probiotics. Monkeys undergoing fecal microbiota transplantation with diarrheal (FMT+D) and normal (FMT+N) feces, as well as those in the probiotic groups. Data in FMT group combined results from both the FMT+D and FMT+N groups. P0W and P6W represent 0 weeks and 6 weeks post-FMT, respectively. Femoral and portal refer to the femoral and portal veins, which are anatomical sites for blood collection. The lines in the graphs indicate the mean values with standard deviation (SD). Statistically significance were determined by t test and indicated by p-values. P ≤ 0.05 (*); P ≤ 0.01 (**); P ≤ 0.001 (***). GIP, glucose-dependent insulinotropic polypeptide; PP, pancreatic polypeptide.

## Slide 12
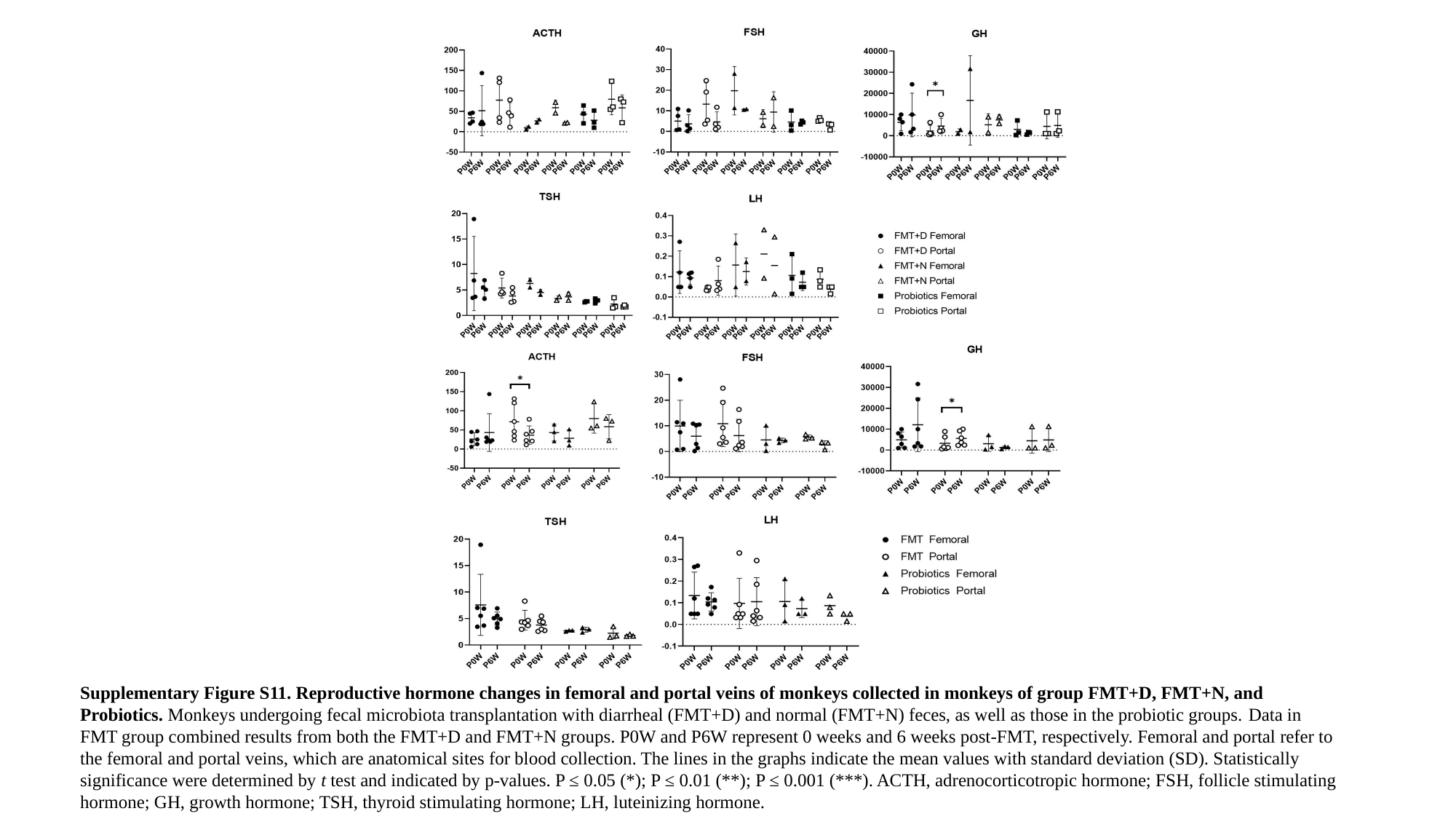

Supplementary Figure S11. Reproductive hormone changes in femoral and portal veins of monkeys collected in monkeys of group FMT+D, FMT+N, and Probiotics. Monkeys undergoing fecal microbiota transplantation with diarrheal (FMT+D) and normal (FMT+N) feces, as well as those in the probiotic groups. Data in FMT group combined results from both the FMT+D and FMT+N groups. P0W and P6W represent 0 weeks and 6 weeks post-FMT, respectively. Femoral and portal refer to the femoral and portal veins, which are anatomical sites for blood collection. The lines in the graphs indicate the mean values with standard deviation (SD). Statistically significance were determined by t test and indicated by p-values. P ≤ 0.05 (*); P ≤ 0.01 (**); P ≤ 0.001 (***). ACTH, adrenocorticotropic hormone; FSH, follicle stimulating hormone; GH, growth hormone; TSH, thyroid stimulating hormone; LH, luteinizing hormone.

## Slide 13
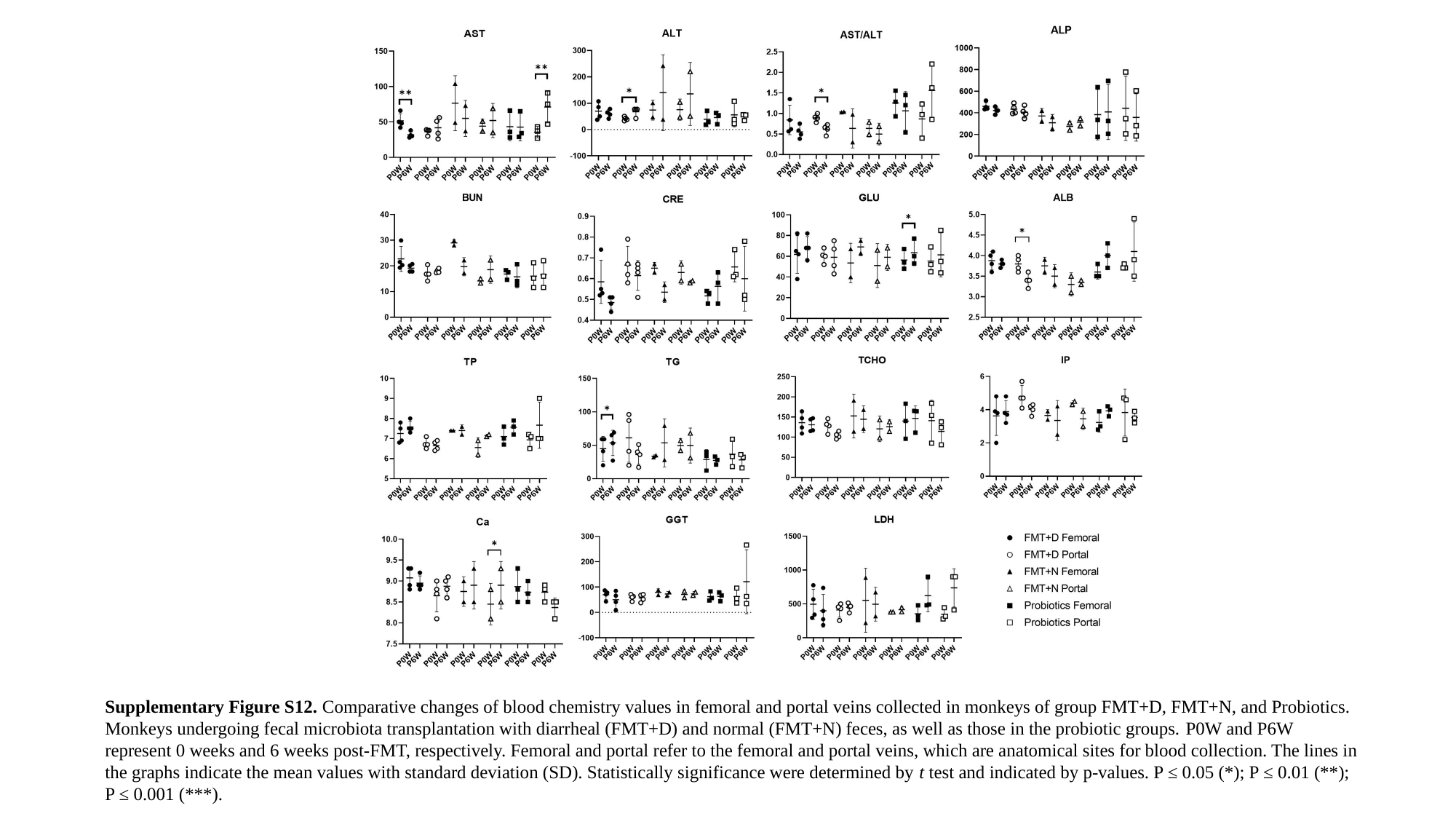

Supplementary Figure S12. Comparative changes of blood chemistry values in femoral and portal veins collected in monkeys of group FMT+D, FMT+N, and Probiotics. Monkeys undergoing fecal microbiota transplantation with diarrheal (FMT+D) and normal (FMT+N) feces, as well as those in the probiotic groups. P0W and P6W represent 0 weeks and 6 weeks post-FMT, respectively. Femoral and portal refer to the femoral and portal veins, which are anatomical sites for blood collection. The lines in the graphs indicate the mean values with standard deviation (SD). Statistically significance were determined by t test and indicated by p-values. P ≤ 0.05 (*); P ≤ 0.01 (**); P ≤ 0.001 (***).
